# Genome plasticity in *Paramecium bursaria* revealed by population genomics

**DOI:** 10.1186/s12915-020-00912-2

**Published:** 2020-11-30

**Authors:** Yu-Hsuan Cheng, Chien-Fu Jeff Liu, Yen-Hsin Yu, Yu-Ting Jhou, Masahiro Fujishima, Isheng Jason Tsai, Jun-Yi Leu

**Affiliations:** 1 Genome and Systems Biology Degree Program, Academia Sinica and National Taiwan University, Taipei, 106 Taiwan; 2 Institute of Molecular Biology, Academia Sinica, 128 Sec. 2, Academia Road, Nankang, Taipei, 115 Taiwan; 3 Biodiversity Research Center, Academia Sinica, Taipei, 115 Taiwan; 4 Graduate School of Sciences and Technology for Innovation, Yamaguchi University, Yamaguchi, 753-8512 Japan

**Keywords:** Ciliate, *Paramecium*, Copy number variation, Programmed DNA rearrangement, Comparative genomics, Minichromosomes

## Abstract

**Background:**

Ciliates are an ancient and diverse eukaryotic group found in various environments. A unique feature of ciliates is their nuclear dimorphism, by which two types of nuclei, the diploid germline micronucleus (MIC) and polyploidy somatic macronucleus (MAC), are present in the same cytoplasm and serve different functions. During each sexual cycle, ciliates develop a new macronucleus in which newly fused genomes are extensively rearranged to generate functional minichromosomes. Interestingly, each ciliate species seems to have its way of processing genomes, providing a diversity of resources for studying genome plasticity and its regulation. Here, we sequenced and analyzed the macronuclear genome of different strains of *Paramecium bursaria*, a highly divergent species of the genus *Paramecium* which can stably establish endosymbioses with green algae.

**Results:**

We assembled a high-quality macronuclear genome of *P*. *bursaria* and further refined genome annotation by comparing population genomic data. We identified several species-specific expansions in protein families and gene lineages that are potentially associated with endosymbiosis. Moreover, we observed an intensive chromosome breakage pattern that occurred during or shortly after sexual reproduction and contributed to highly variable gene dosage throughout the genome. However, patterns of copy number variation were highly correlated among genetically divergent strains, suggesting that copy number is adjusted by some regulatory mechanisms or natural selection. Further analysis showed that genes with low copy number variation among populations tended to function in basic cellular pathways, whereas highly variable genes were enriched in environmental response pathways.

**Conclusions:**

We report programmed DNA rearrangements in the *P*. *bursaria* macronuclear genome that allow cells to adjust gene copy number globally according to individual gene functions. Our results suggest that large-scale gene copy number variation may represent an ancient mechanism for cells to adapt to different environments.

**Supplementary information:**

The online version contains supplementary material available at 10.1186/s12915-020-00912-2.

## Background

Copy number variation (CNV) resulting from segmental DNA duplications or deletions (usually ≥ 50 base pairs, bp) represents a crucial source of genetic variation contributing to phenotypic diversity [1, 2]. For decades, most studies of phenotypic diversity focused on single nucleotide polymorphisms (SNPs), whereas the effects of CNV remain understudied [3]. However, owing to improved sequencing techniques, the prevalence and importance of CNV in model organisms are gradually being revealed [4–8].

Most large-scale studies on CNV have been conducted on human populations, which show that de novo CNV frequently occurs in human genomes [9, 10] and accounts for 17.7% of altered gene expression among genes associated with CNV or SNPs [11]. Moreover, CNV has been linked to a broad range of genetic diseases and complex traits [10, 12]. More recently, the phenotypic outcomes of CNV have also been investigated in other organisms, including domestication traits in animals and plants [13], chemical or disease resistance in insects and plants [14, 15], and environmental adaptation in microorganisms [3, 16]. These studies provide examples of how organismal phenotypes are influenced by CNV. However, it remains unclear to what extent CNV can be tolerated in a typical genome and if organisms systematically utilize CNV to adjust their physiologies under different environments.

Ciliates are excellent models for studying tolerance to and regulation of CNV [17–21]. A unique feature of ciliates is their nuclear dimorphism, by which two types of nuclei are present in the same cytoplasm and serve different functions throughout the life cycle [22]. The micronucleus (MIC) contains the diploid and transcriptionally silent germline genome, whereas the macronucleus (MAC) harbors the polyploid and transcriptionally active somatic genome. During asexual reproduction, the MIC undergoes typical mitosis and the MAC divides by amitosis, a type of nuclear division that does not involve spindle fibers. Amitotic nuclear division does not precisely segregate duplicated chromosomes, probably allowing ciliates to have a highly plastic MAC genome. To date, the underlying mechanism of amitotic chromosome segregation has remained largely uncharacterized. In each round of sexual reproduction, only the genetic material in the MIC is passed on to the progeny. The existing MAC is degraded after fertilization and a new MAC develops from the endoduplicated zygote. During this developmental period, a series of large-scale programmed DNA rearrangement events occur including DNA amplification, chromosome breakage, and excision of internal eliminated sequences (IESs) and repeated sequences. These rearrangements result in the formation of minichromosomes that can further increase the flexibility of CNV regulation.

Various types of genome rearrangements have been identified during MAC development in different clades of ciliates [23–26]. For instance, in *Tetrahymena thermophila*, five pairs of chromosomes are broken at specific sites and telomeric repeats are added de novo [27–30], resulting in the formation of approximately 181 specific minichromosomes averaging 68 copies each [31–33]. In *Paramecium tetraurelia*, the chromosomes undergo precise elimination of IESs and imprecise removal of repeated elements and transposons, and they are highly amplified to around 800 copies [34]. After DNA elimination, the ends are either joined by non-homologous end joining (NHEJ) or telomeres are added de novo to form minichromosomes. For both *T*. *thermophila* and *P*. *tetraurelia*, minichromosomes are maintained at constant copy numbers after conjugation. However, it has been observed in *P*. *tetraurelia* that minichromosomes might be fragmented by DNA damage and become shorter during clonal aging [35]. In *Oxytricha trifallax*, after the IESs have been removed, its chromosomes are extensively fragmented and unscrambled to form thousands of nanochromosomes that typically carry only one to eight genes [36, 37]. Nanochromosome copy number can be further adjusted by means of a maternal RNA-mediated mechanism during MAC differentiation [20, 21, 38].

*Paramecium bursaria* is one of only two species in the genus *Paramecium* that harbor algal endosymbionts [39, 40]. Based on a phylogenetic tree constructed from *Paramecium* 18S rRNA sequences with *T*. *thermophila* as outgroup, *P*. *bursaria* is the most diverged species since the most common *Paramecium* ancestor [41], which may explain why *P*. *bursaria* cell physiology is so distinct from other *Paramecium* species. In the wild, most *P*. *bursaria* cells stably harbor several hundred algal cells in the cytoplasm [42]. Each algal cell is engulfed by a host-derived perialgal vacuole (PV) membrane that becomes attached to the host plasma membrane [43, 44]. Different species of algal endosymbionts have been identified in different strains of *P*. *bursaria* [45], suggesting that these strains may have evolved a preference for or compatibility with specific green algae. Thus, *P*. *bursaria* strains and their endosymbiotic algae represent a useful system for understanding how stable endosymbiosis is initiated and maintained.

To facilitate genomic analyses of *P*. *bursaria*, we sequenced and annotated the functional macronuclear genome of *P. bursaria*. Unlike *T*. *thermophila* in which individual minichromosomes are maintained in similar copy numbers [31, 32], we observed extensive CNV at both chromosome and gene levels. By comparing the genomes of different *P*. *bursaria* strains, we reveal that minichromosomes maintained in consistent copy numbers among strains are enriched with housekeeping genes, whereas those carrying environmental response genes exhibit pronounced CNV among strains. Our results suggest that *P*. *bursaria* exhibits high plasticity and tolerance to gene copy number variation, which may facilitate cell adaptation to different environments.

## Results

### A global view of the *Paramecium bursaria* macronuclear genome

**Fig. 1:**
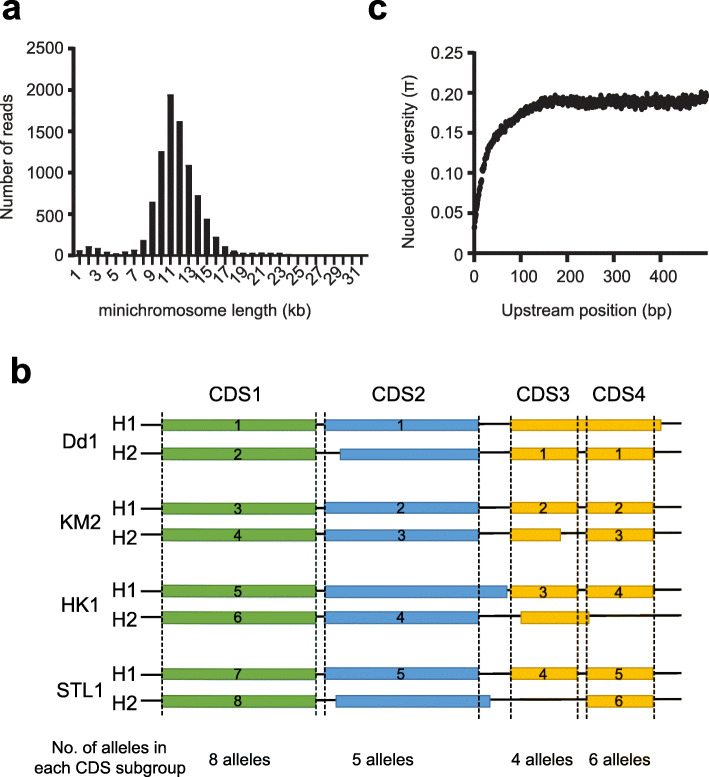
Identification of the functional gene set and the general features of intronic and intergenic regions. **a** The length distribution of full-length minichromosomes from PacBio sequencing. We identified about 9000 reads with telomeric repeats (C_4_A_2_) n or (T_2_G_4_) n at both ends of a single read, which represent full-length minichromosomes. **b** Categorization of alleles at the same locus into different CDS subgroups using population genomics data. Alleles sharing the same sequence positions for the start and stop sites were classified into a CDS subgroup. For each locus, we chose the subgroup with the highest number of alleles to identify the functional allele. If the difference between the allele numbers of the top two subgroups was equal to or less than one, we chose the subgroup with a longer coding region (see the “Methods” section). **c** Mean nucleotide diversity of the sequences upstream of the coding region. Position 1 represents the first base pair upstream of the translation start site

To assemble a macronuclear reference genome of *P*. *bursaria*, we isolated macronuclei from an aposymbiotic strain (i.e., cells without endosymbionts), Dd1, and sequenced the genomic DNA using both Illumina and PacBio sequencing platforms (Additional file 1: Fig. S1a). From the PacBio long-read data, we noticed that most full-length minichromosomes (with telomeric repeats at both ends) were around 8 to 16 kb in length (Fig. 1a). Moreover, even the minichromosomes with similar gene contents had highly variable breakage sites. To reduce the complexity of the reference genome, we decided to use the overlapping regions between different minichromosomes and long reads to assemble artificial long contigs (see the “Methods” section). Among 597 contigs of the initial 30.1 Mb assembly, 129 contigs shared greater than 70% sequence identity to other contigs, perhaps due to highly divergent haplotypes. We removed redundant parts of these contigs, preserving only regions containing unique coding sequences (CDSs), and renamed them as regions of internal structural variation (ISVs; Additional file 1: Fig. S1b). After manually examining the alignment, we further scaffolded 55 contigs by means of end sequence homology. The final assembly included 413 contigs and 102 ISVs totaling 26.8 Mb (Table 1). The GC content of the genome (28.8%) is similar to that reported for other *Paramecium* species [46, 47]. Combining the information from the size of our assembled MAC genome and previous microspectrophotometry data [52], we estimated that the average gene copy number is around 6000 in MACs.

**Table 1:** Comparison of different ciliate macronuclear genomes

	*Paramecium bursaria*	*Paramecium caudatum*^a^	*Paramecium tetraurelia*^b^	*Tetrahymena thermophila*^c^	*Oxytricha trifallax*^d^
Genome size (Mb)	26.8	30.5	72.1	103	55.4
Sequencing platform	PacBio and Illumina	Illumina	Shotgun sequencing and Illumina	Shotgun sequencing and Illumina	PacBio and Illumina
No. of contigs	515 (413 + 102 ISV)	1202	697	1158	19,152
Size distribution of chromosomes observed using pulsed-field electrophoresis (Kb)	6–90^e^	50–750^f^	50–1000^g^	21–1500^f^	0.4–40^h^
N50 (Kb)	100	312.9	413	520	3.5
Longest contig (Kb)	250	793	980	2216	66
Genomic GC content (%)	28.8	28.2	28.0	22.0	31.0
Gene number	15,101	18,673	40,460	26,996	18,400
Average gene length (bp)	1513	1458	1427	2400	1839

*ISV* internal structure variation region

^a^Taken from [46]

^b^Taken from [47, 48]

^c^Taken from [31, 49]

^d^Taken from [37]

^e^This study

^f^Taken from [50]

^g^Taken from [47]

^h^Taken from [51]

An initial analysis revealed that the sequence diversity of the two haplotypes was high in our sequenced genome, which may result in inaccurate gene annotation due to misassignment of two alleles. We performed haplotype phasing to construct both haplotypes and then carried out gene annotation by combining de novo gene prediction and RNA sequencing (Additional file 1: Fig. S1a, Additional file 2: Table S1, and Methods). We found that sequence divergence between the two haplotypes was on average 2.5%, which is significantly higher than reported for several well-studied eukaryotes [53–55]. We annotated about 15,600 CDSs from each haplotype and ~ 40% (6273/15,601) either exhibited different start or/and stop sites between two alleles or were only present in one haplotype (Fig. 1b), suggesting that a large proportion of the proteome may be truncated and/or have lost function.

To construct a functional gene set, we further sequenced the macronuclear genomes of another three genetically divergent *P*. *bursaria* strains (KM2, HK1, and STL3). If we detected similar gene structures or alleles among these different strains, they would more likely represent functional genes/alleles (Fig. 1b and the “Methods” section). The genomes of these three strains exhibited a sequence diversity ranging from 2.1 to 4.0% relative to the Dd1 reference genome (Additional file 2: Table S2), providing useful population data for annotating the functional genome. In total, 15,101 genes were classified into our functional gene set. To evaluate the completeness of our assembled genome, we examined it using the Core Eukaryotic Gene Mapping Approaches (CEGMA) database and identified 225 of 248 (91%) eukaryotic core genes with BLASTP [56]. Compared to the 220–230 core genes identified in the *Tetrahymena*, *Oxytricha*, and other *Paramecium* genomes [31, 36, 46, 47], this outcome indicates that our assembly is comparably complete.

Similar to other *Paramecium* species [46, 47, 57], the *P*. *bursaria* genome has extremely short intergenic regions and introns (Additional file 2: Table S3, Additional file 2: Fig. S1d and S1e), suggesting that the transcriptional regulation and splicing machineries of *P*. *bursaria* deviate considerably from non-ciliate organisms. Functional parts of noncoding regions are typically more conserved than nonfunctional sequences when different organismal populations are compared [58, 59]. We used population data to calculate the nucleotide diversity (π) of the noncoding regions upstream of the translation start sites of individual genes. We found that sequence diversity gradually increased from the translation start site and reached a plateau after 150 bp (Fig. 1c). These data suggest that regulatory elements are mainly located within 50 bp upstream of the coding region, which in *P*. *bursaria* is more compact than in other well-studied eukaryotic organisms [59]. We also examined all the introns and discovered that the 5′ (GT) and 3′ (AG) splice sites remained conserved (Additional file 1: Fig. S2 [60]). Nonetheless, of the 92 splicing-related genes in *S*. *cerevisiae*, we only identified 66 orthologs in our assembled *P*. *bursaria* genome (Additional file 3: Table S4). The missing genes include some important components that are essential for yeast viability (*AAR2*, *CWC25*, *LSM8*, *PRP24*, *PRP39*, *PRP42*, *SNU114*, and *SPP2*) and that are shared between yeast and human cells.

### Protein family expansion and gene duplication in *Paramecium bursaria*

**Fig. 2:**
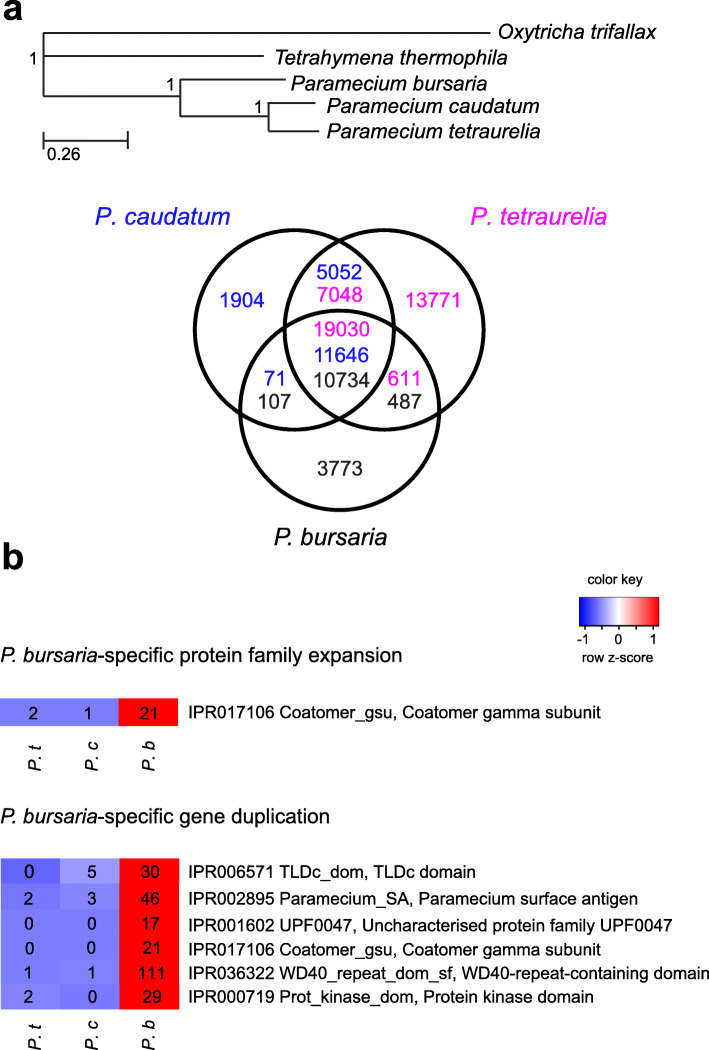
Comparison of different *Paramecium* genomes. **a** Seventy-five percent of *P*. *bursaria* genes have orthologs in other *Paramecium* species. The phylogenetic tree was constructed using the core gene set of ciliates listed in Table 1 (see the “Methods” section). Numbers beside each node are posterior probability values obtained from Bayesian posterior probability. The scale bar represents the number of amino acid substitutions per site. The Venn diagram shows the number of orthologous genes shared among three *Paramecium* species. Orthologous genes were defined by comparing all protein sequences using OrthoFinder [61]. Different colors indicate the gene number of each species. The gene number of *P*. *tetraurelia* is more than that of other species due to the species having undergone two whole-genome duplication events [47]. **b** Heat map of the protein families and duplicated genes enriched in the *P*. *bursaria* genome. The gene numbers in each protein family/group of paralogs of each species are shown. The proportion of genes belonging to each protein family/group of paralogs to the whole genome gene number in each species was converted into a *z*-score by z-transformation (*z* = (*x* − μ)/σ, where *μ* is the population mean and *σ* is the population standard deviation). A two-proportional test was conducted and proteins or gene families are shown having a *p* value ≤ 0.05 after Benjamini-Hochberg adjustment

Among the known *Paramecium* species, *P*. *bursaria* is the most divergent lineage [41] and one of only two species that can stably establish endosymbiotic relationships with *Chlorella* spp. To gain insights into the specific physiology of *P*. *bursaria*, we compared the functional gene set of *P*. *bursaria* with those of another two sequenced *Paramecium* genomes, *P*. *caudatum*, and *P*. *tetraurelia*. We found that 75% (11,328/15,101) of *P*. *bursaria* genes have orthologs in these other *Paramecium* species (Fig. 2a). Among 3773 *P*. *bursaria*-specific genes, gene ontology (GO) terms related to phosphorelay signal transduction, drug transmembrane transport, and vesicle-mediated transport were significantly enriched (Additional file 2: Table S5). Genes related to these GO terms are also known to be expanded in *T*. *thermophila* relative to animals and yeast [31]. The observed enrichment suggests that some existing pathways may have been further modified in *P*. *bursaria* to innovate lineage-specific phenotypes.

To investigate if specific protein families are expanded in *P*. *bursaria*, we compared the gene numbers of all known protein families between *P. bursaria*, *P*. *caudatum*, and *P*. *tetraurelia*. Only one protein family was specifically enriched in the *P*. *bursaria* genome (Fig. 2b); the coatomer complexes coat membrane-bound vesicles and play an important role in vesicle transportation [62, 63]. Previous studies in *P. bursaria* have shown that during the establishment of endosymbiosis, green algae are always coated with a PV membrane and transported beneath the host cell cortex [64, 65]. Moreover, pathogens are known to hijack the coatomer-mediated pathway to enter and populate host cells [66]. Expansion of the coatomer family in *P*. *bursaria* probably represents a co-evolutionary event specific to algal endosymbiosis.

Gene duplication has also been suggested to contribute to specific adaptation during organism evolution [67, 68]. We further analyzed lineage-specific enrichment of gene duplication using OrthoFinder [61]. We found that six gene families are highly duplicated only in *P. bursaria* (Fig. 2b). The WD40 domain occurs in a large group of gene families in eukaryotes, and WD40 domain-containing proteins often function as a scaffold in various cellular pathways. Moreover, WD40 domain-containing proteins have been reported to be involved in legume-*Rhizobium* symbioses [69]. TLDc domain-containing proteins primarily function as antioxidants to protect cells from reactive oxygen species (ROS) [70, 71], and algal endosymbiosis has been shown to generate excess ROS in different hosts [72]. Expansion of the TLDc domain-containing gene in *P*. *bursaria* may be an adaptation to such oxidative stress. Expression of *Paramecium* surface antigen domain-containing genes is often induced by environmental changes [73, 74], but it remains unclear why *Paramecium* surface antigen-containing, UPF0047 domain-containing, and protein kinase domain-containing genes are highly duplicated in *P*. *bursaria*.

### High heterogeneity of macronuclear minichromosome structures

**Fig. 3:**
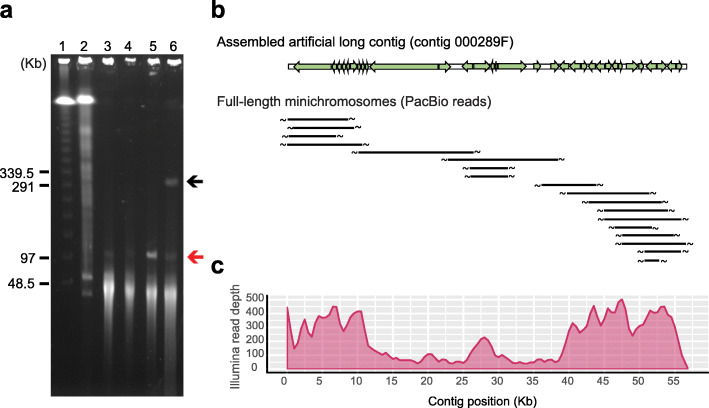
High heterogeneity of minichromosomes leads to non-uniform gene dosage. **a** The length distributions of minichromosomes are similar between young and old cells. Pulsed-field gel electrophoresis (PFGE) patterns of the aposymbiotic cells of Dd1 (lane 3), KM2 (lane 4), DK1 (lane 5), and the symbiotic cells of newly formed progeny DK2 (lane 6). Both DK1 and DK2 are the progeny of Dd1 and KM2 and they are about 250 and 17 generations old, respectively. The red arrow indicates the mitochondrial DNA and the black arrow indicates the *Paramecium bursaria Chlorella* virus-1 (PBCV-1) DNA of the green algae (see also Fig. S3 [see Additional file 1]). Lane 1 is the Lambda DNA ladder. Lane 2 is the control sample of the *T*. *thermophila* BII minichromosomes to show that full-length minichromosomes were maintained during our sample preparation. **b** An example showing that minichromosome copy numbers are highly variable. In this case, long reads with telomere repeats at both ends from the PacBio data have been mapped to an assembled artificial long contig 000289F. Each read represents an intact minichromosome in the MAC. Green arrows represent the location and orientation of predicted coding sequences. The ~ symbols represent telomeres of minichromosomes. **c** The read depth of the Illumina data is highly correlated with PacBio data (Spearman correlation *ρ* = 0.75, *p* value < 0.0001). Sequencing coverage is calculated from the depth of Illumina reads. The distribution of read depth has been drawn using a 1-kb interval and 0.5-kb sliding window. Genomic DNA of the Dd1 strain was used to generate the PacBio and Illumina data

Global DNA rearrangements during the development of new MACs are a peculiar feature of ciliates. Both precise elimination of IESs and imprecise removal of repeat regions have been reported for the *Paramecium* genus [34, 75]. After imprecise elimination of DNA, chromosome ends are either joined with other DNA fragments or telomeric repeats are added de novo. Taking advantage of long-read sequencing, we identified about 9000 reads with telomeric repeats (C_4_A_2_) n or (T_2_G_4_) n at both ends of a single read, which represent full-length minichromosomes. The size distribution of minichromosomes is concordant with the chromosome sizes observed in the pulsed-field gel electrophoresis (Fig. 1a and Fig. 3a; Additional file 1: Fig. S3 [76, 77]). Moreover, the telomere addition sites were highly variable even for minichromosomes carrying similar gene contents (Fig. 3b; Additional file 1: Fig. S3d), which could further contribute to non-uniform gene dosage. Alternative telomere addition sites have been observed in other *Paramecium* species [78–80]. However, the *P*. *bursaria* macronuclear genome exhibited a quantitative difference in that chromosome breakage sites are much denser, resulting in shorter minichromosomes and higher variation in copy number.

To rule out the possibility that this pattern is an artifact of the PacBio sequencing platform, we mapped the Illumina reads that ended with telomeric repeats (*n* = 99,428) against the MAC genome assembly and found that the read depth was indeed correlated with those inferred from PacBio long reads (*n* = 144,615; Spearman correlation *ρ* = 0.57, *p* < 0.0001, Methods, Additional file 1: Fig. S4 and Additional file 4: Table S6). Moreover, the non-uniform pattern of gene dosage was further supported by the read depth of total Illumina reads (Spearman correlation *ρ* = 0.75, *p* < 0.0001, Fig. 3c). These results indicate that alternative telomere addition occurs throughout the macronuclear genome of *P*. *bursaria* (Additional file 1: Fig. S5).

Previously, clonal aging has been shown to cause random fragmentations of minichromosomes in *P*. *tetraurelia* [35]. Although clonal aging has not been reported in *P*. *bursaria*, there was a possibility that the short and highly variable minichromosomes observed in our strains were the outcome of long-term asexual propagation. To investigate this possibility, we examined the MAC genomes of two young F1 diploids, DK1 and DK2, generated from crossing the Dd1 and KM2 strains. The DK1 cells had been asexually propagated for about 250 generations and their endosymbionts had been removed. DK2 cells were immature young cells (about 17 generations old) that still harbor green algae. The pulsed-field electrophoresis data showed that both young and old cells exhibited similar length distributions of minichromosomes (Fig. 3a; Additional file 1: Fig. S3d). Moreover, whole-genome sequencing data revealed that young DK2 cells also exhibit highly variable breakage sites (Additional file 1: Fig. S6). These data rule out the possibility that the high heterogeneity of minichromosome structures observed in our *P*. *bursaria* strains is caused by clonal aging. Moreover, it indicates that variable chromosome breakage occurs during or soon after the development of new MACs in sexual reproduction.

### Extensive copy number variation in the macronuclear genome

**Fig. 4:**
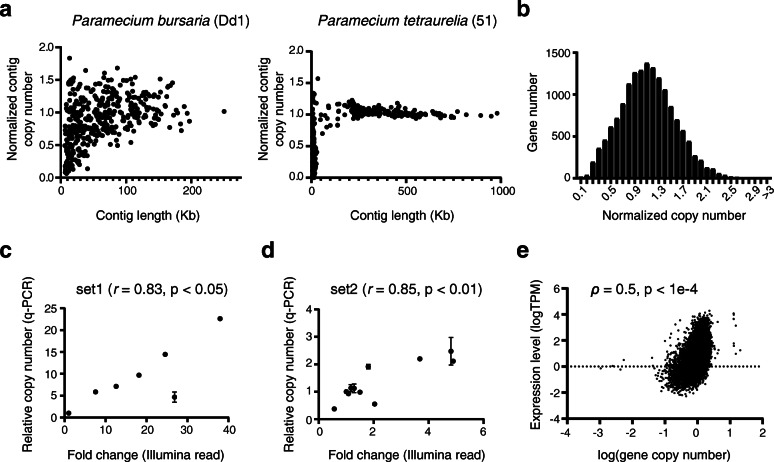
The *P*. *bursaria* genome shows extensive copy number variation. **a** Scatter plot of contig length and normalized coverage of each contig. The copy numbers of contigs are highly variable in *P*. *bursaria*, but remain consistent in *P*. *tetraurelia*. The short contigs with lower coverage in *P*. *tetraurelia* are either the unassembled ends of scaffolds or AT-rich reads underrepresented in the sequencing libraries [81]. **b** Histogram showing the number of genes with different copy numbers in the genome. About one third of *P*. *bursaria* genes have copy numbers larger than 1.5-fold or smaller than 0.67-fold of the average copy number of the genome. Data in **a** and **b** are from Dd1. **c**, **d** Validation of gene dosage estimated from whole-genome sequencing by qPCR. Seven genes that cover the full range of copy number variation in KM2 were examined in **c**, and 11 genes that cover the lower end of copy number variation in Dd1 were examined in **d**. All values were normalized to the same internal control CDS 125F.32. Error bars represent the standard error (*n* ≥ 3). **e** Scatter plot showing that gene expression is positively correlated with gene copy number (*n* = 15,205). The Spearman correlation coefficient (*ρ*) and *p* value are shown

The copy number of individual minichromosomes in *T*. *thermophila* and *P*. *tetraurelia* is uniform [31, 81]. When we analyzed the average read depth of assembled contigs, a majority of them fell within a range of 0.67-fold to 1.5-fold of the genome average (Fig. 4a; Additional file 1: Fig. S6a). In contrast, the average read depth of *P*. *bursaria* contigs was distributed across a broader range (Fig. 4a). CNV could be further depicted at the gene level. In the Dd1 reference genome, about one third of *P*. *bursaria* genes (5249/15,101 = 35%) had copy numbers larger than 1.5-fold or smaller than 0.67-fold of the average copy number of the genome (Fig. 4b). Similar trends were also observed in other *P*. *bursaria* strains, including the newly generated DK2 cells (Additional file 1: Fig. S6b).

To confirm that CNV was not due to a bias in mapping or sequencing processes, we selected two sets of genes to validate copy numbers by quantitative PCR. The first set comprised genes covering the full range of CNV and the second set represented only genes with lower copy numbers. Our results showed high correlations between quantitative PCR and Illumina sequencing data (Fig. 4c and d), indicating that *P. bursaria* can tolerate a wide range of gene dosages, unlike *T. thermophila* and other sequenced *Paramecium* species.

In other organisms, changes in gene copy number are often associated with alterations in gene expression levels [3, 18, 82]. We observed a positive correlation between mRNA abundance and gene copy number in *P*. *bursaria* (Fig. 4e), suggesting that in addition to transcriptional regulation, the observed CNV also contributes to variation in gene expression.

### Gene copy number variation is correlated with the gene function

**Fig. 5:**
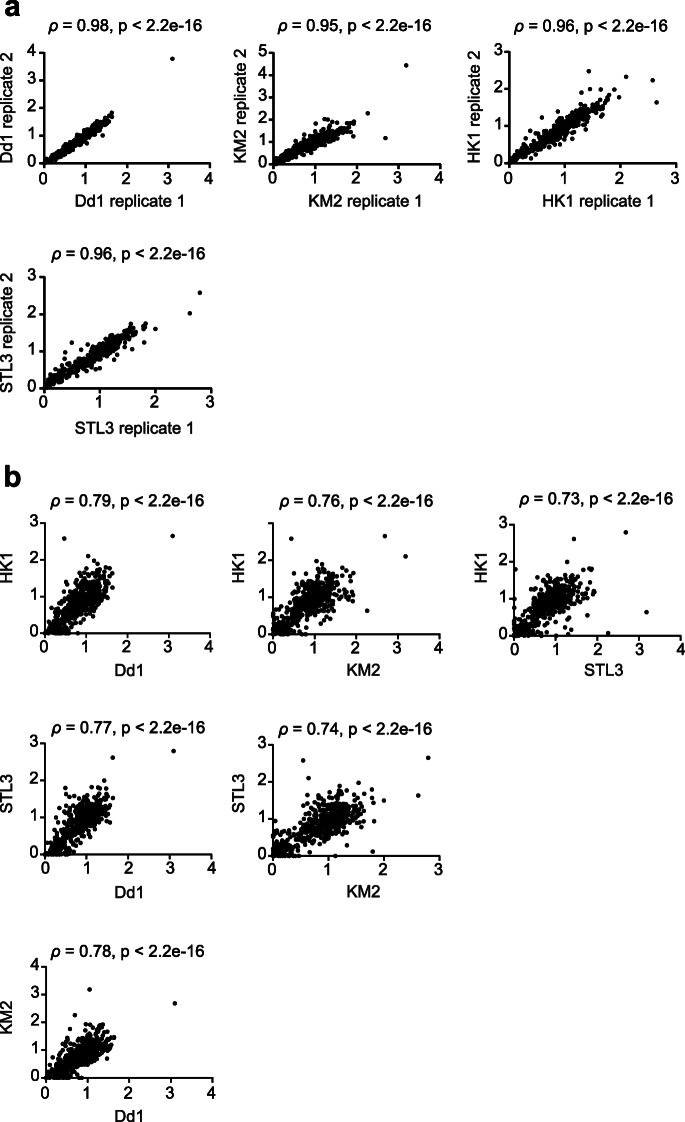
The copy numbers of contigs are conserved between replicates and different strains. **a** Contig copy numbers are strongly correlated between two biological repeats of the same strain collected at different time points. **b** The correlation of contig copy numbers between different strains is still high, but lower than that between two replicates. The *x*- and *y*-axes represent the read depth of each contig normalized to both contig length and whole-genome coverage for the respective strain. The Spearman correlation coefficient (*ρ*) and *p* value are shown

Random minichromosome assortment has been suggested to be the primary mechanism for chromosome segregation in MAC during asexual reproduction of ciliates. Amitosis is poorly characterized and it remains unclear if the patterns of CNV can be stably maintained during amitosis. To address this question, we sequenced and compared genomic DNA from different replicate populations of the same strains. Contig copy numbers were highly correlated between replicates even though some of the populations had diverged for more than 100 generations (Fig. 5a; Additional file 2: Table S9). We observed similar patterns when we compared the copy number of individual genes (Additional file 2: Table S7). The slightly reduced correlation is probably due to the small size of genes that makes the data noisier. These data suggest that CNV patterns can be stably maintained during asexual reproduction.

**Fig. 6:**
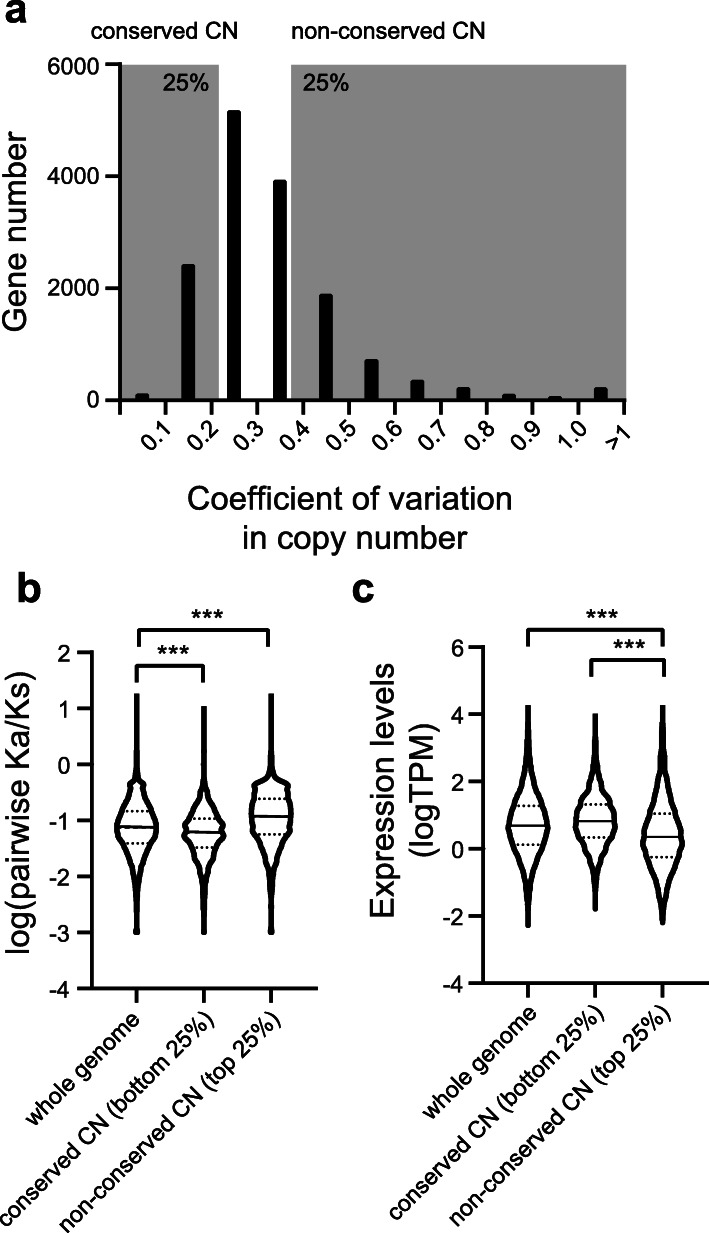
Copy number variation is correlated with evolutionary rate and expression levels. **a** The distribution of coefficient of variation (CV) in copy number for each gene using population genomics data from four strains. The top and bottom 25% of genes in terms of CV (labeled in gray) are categorized into non-conserved and conserved copy number (CN) groups, respectively. **b** Pairwise Ka/Ks values are significantly lower in the conserved CN group and higher in the non-conserved CN group when compared to those of the whole genome. **c** Gene expression of the non-conserved CN group is significantly lower than that of the conserved CN group. ***p value < 0.0001, two-tailed Mann-Whitney *U* test

More interestingly, when we compared the copy numbers of assembled contigs between genetically divergent strains, the correlation coefficients remained high but they were significantly lower than those obtained from within-strain comparisons (Fig. 5b) (*p* < 0.005, one-tailed Mann-Whitney *U* test). One possible explanation for this outcome is that genes related to basic cellular pathways have conserved CNV patterns between different strains, but genes related to strain-specific or condition-specific phenotypes have more variable CNV patterns. To test this hypothesis, we calculated between-strain copy number variability for each gene using the genomic data from four different *P*. *bursaria* strains (Fig. 6a). The top (non-conserved group) and bottom (conserved group) 25% of genes in terms of their CNV were chosen for subsequent analyses.

First, we examined the relative evolutionary rate (Ka/Ks) of these two groups of genes. In general, genes involved in basic cellular functions or occupying a central position in regulatory networks have lower evolutionary rates, whereas genes involved in strain-specific or condition-specific phenotypes evolve rapidly [83–85]. We found that the evolutionary rate of the conserved group of genes was significantly lower than that of the whole genome gene set (*p* < 0.0001, two-tailed Mann-Whitney *U* test, Fig. 6b). In contrast, the non-conserved group had a significantly higher Ka/Ks value than the whole genome gene set (*p* < 0.0001, two-tailed Mann-Whitney *U* test). Second, we performed GO enrichment analysis. The conserved group tended to be involved in general pathways, such as basic cellular processes or cellular component biogenesis (Additional file 2: Table S8), whereas the non-conserved group was enriched for functions related to environmental responses. Finally, we compared the gene expression levels of these two groups of genes. Housekeeping genes are stably expressed, whereas genes related to condition or strain adaptation are often only induced under specific conditions [86]. Indeed, we found that the non-conserved group presented significantly lower expression than either the conserved group or the whole genome dataset (*p* < 0.0001, two-tailed Mann-Whitney *U* test, Fig. 6c). Together, our data suggest that *P. bursaria* has evolved the ability to adjust gene copy number according to gene function.

## Discussion

Due to high heterozygosity and structural variation of the two haplotypes in the diploid cell, it is notoriously difficult to annotate correctly the macronuclear genome of *P. bursaria*. So far, no homozygous *P*. *bursaria* diploid lines have been established since *P*. *bursaria* does not undergo autogamy (an autofertilization process) like other *Paramecium* species [87]. In a recent study reporting a draft of the *P*. *bursaria* genome [88], only about 7000 genes had orthologs in closely related *P. caudatum*, despite 17,226 genes being annotated. By combining population genomic data and gene expression profiles, we have established a functional genome of *P*. *bursaria* that contains 15,101 genes, 72% (10,841/15,101) of which share orthologs with *P*. *caudatum* (Fig. 2a). Our significantly improved annotation allowed us to perform more in-depth genome comparisons between *Paramecium* species and for intraspecific populations.

*P. bursaria* has very short intergenic and intronic regions compared to other characterized eukaryotic genomes [31, 59, 89, 90]. Our nucleotide diversity analysis indicated that the intergenic sequences are most conserved in the first 50 bp upstream of the translation start site (Fig. 1c), suggesting that transcription regulatory elements are mainly located within this small region. Compared to other eukaryotic organisms that possess sophisticated regulatory elements in long intergenic regions, the functional flexibility of *cis*-regulatory elements in *P*. *bursaria* may be restricted. This raises the possibility that the CNV adjustment provides another layer of flexibility for gene regulation. Despite the 5′ and 3′ splice sites being highly conserved in our annotated genes, the *P. bursaria* genome only contains orthologs of 72% and 79% of splicing-related genes in yeast and plants, respectively. Moreover, several essential splicing-related genes shared by both yeast and human cells are missing from the *P. bursaria* genome. We extended our analysis to other ciliate genomes, including other *Paramecium* species and *T*. *thermophila*, and confirmed that these splicing-related genes are absent from these ciliates. This outcome indicates that these essential proteins have been substituted by other uncharacterized functional orthologs or the splicing machinery has been extensively modified in ciliates. Since every *P*. *bursaria* gene contains 2.4 introns on average (Additional file 2: Table S3), the splicing machinery is not a “reduced” form of spliceosomes as observed in intron-poor eukaryotes [91] even if it significantly diverges from that which operates in other eukaryotic cells. Ciliates provide an interesting system for studying the evolution of spliceosomes.

We identified 102 internal structural variations (ISVs) that account for 2% of our genome assembly, and 238 genes were annotated from ISVs. These ISVs were generated from assembled contigs that shared a significant portion of homologous regions with other contigs (Additional file 1: Fig. S1b). To avoid preserving redundant contigs (and CDSs) in the assembly, we only retained the unique regions that contained CDSs. There are two possible origins for those ISVs. First, an ISV may represent one of a pair of homologous chromosomes carrying some highly diverged sequences. In this scenario, the ISV would only have one haplotype in the genome. We counted 71 ISVs that belong to this type. In contrast, 31 ISVs were observed to have two haplotypes, suggesting that the redundant contigs may be isoforms generated through different DNA rearrangements of the same micronuclear chromosome. Alternative DNA rearrangements have been observed in other ciliates. For instance, an alternative DNA deletion of the M element in *T*. *thermophila* results in two minichromosomes with different lengths in the MAC [92]. Studies in *O*. *trifallax* have further shown that extensive alternative DNA rearrangements can generate minichromosomes with different gene products [93–95]. Whether alternative DNA rearrangements have specific regulatory functions in *P*. *bursaria* remains to be addressed.

Our data reveal intricate chromosome breakage patterns in the MAC, which further contribute to highly variable gene dosages in the *P*. *bursaria* genome. Although variable gene dosages were also observed in *Spirotrich*, the composition and generation of macronuclear chromosomes are different from *P*. *bursaria*. For instance, the majority of the nanochromosomes of *O*. *trifallax* contain only one gene and the telomere addition sites are primarily located in the intergenic regions [36]. We found most of the minichromosomes in *P. bursaria* contain more than one gene and ~ 30% of the telomere addition sites are located in the coding regions. Interestingly, the cluster analysis indicated that the proportion of genes in clusters was significantly higher in the conserved copy number group (79.0%) compared to random sampling (45.1%, 95% confidence interval 43.4–46.8%, *p* value< 0.001, bootstrapping = 1000, see the “Methods” section). It raises the possibility that conserved genes may be clustered on the same minichromosomes so the copy number can be adjusted together.

In other ciliates, specific motifs have been identified near the chromosome breakage sites only when precise elimination is involved [27–29, 96, 97]. A study of *P*. *tetraurelia* suggested that chromosome breakage sites are often located near repeated elements or transposons, but no specific motif was found [34]. We compared the distributions of telomere addition sites between different *P*. *bursaria* strains and observed significant correlations (Spearman correlation *ρ* = 0.51, *p* < 0.0001 for KM2 and Dd1; *ρ* = 0.54, *p* < 0.0001 for DK1 and Dd1; *ρ* = 0.52, *p* < 0.0001 for KM2 and DK1). It suggests that different strains share some chromosome breakage hotspots and sequence motifs may be involved. Since the breakage sites in *P*. *bursaria* exhibit high heterogeneity, we used genomic regions that have at least four telomere-containing reads to search for specific motifs (see the “Methods” section). Only GA-rich motifs were found to be highly enriched (Additional file 1: Fig. S7 [98]). More experiments are required to validate the cis-regulatory functions of these motifs. Nonetheless, our findings should prompt further investigations of the molecular mechanisms underlying complex chromosome breakage.

**Fig. 7:**
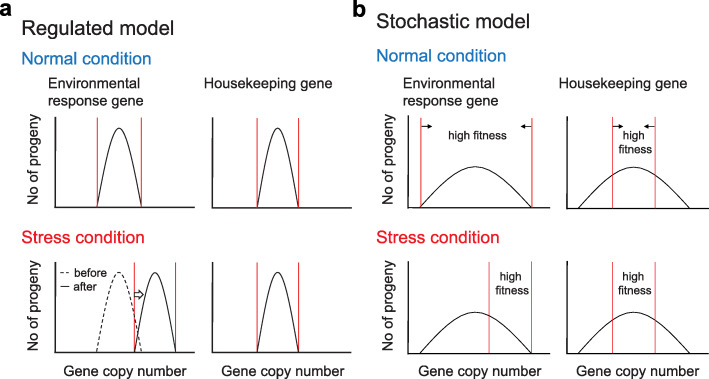
Models explaining how the copy number of genes with different functions is adjusted or selected. **a** In the regulated model, the duplicated minichromosomes distributed to the daughter cells are controlled within a range during amitosis. When cells encounter a stress environment, the copy number of environmental response genes is specifically adjusted to adapt to the new environment. **b** In the stochastic model, the duplicated minichromosomes are randomly distributed to the daughter cells during amitosis and the CNV pattern is shaped by natural selection. The housekeeping genes are more sensitive to dosage variation so only the progeny carrying correct dosages display high fitness and there is no selection for the dosage of environmental response genes under normal conditions. However, cells carrying certain copies of environmental response genes would be selected for in a stress environment

The correlation between gene copy numbers and expression levels reveals the biological impact of CNV, as previously observed in other organisms [11, 13–15, 99]. The distinct evolutionary rates and expression levels between highly variable and lowly variable gene groups further suggest that CNV levels may be adjusted or selected according to gene function. At least two possible scenarios can explain our current observation. First, the copy numbers of individual minichromosomes are always controlled in a specific range according to their functions. When the cells encounter environmental changes, the copy numbers of minichromosomes required for adapting to the new environment are also adjusted (Fig. 7). In *Oxytricha*, an RNA-mediated mechanism has been shown to regulate chromosome copy number during sexual reproduction [21]. A similar but modified mechanism may be used by the *P. bursaria* cells to adjust CNV during asexual reproduction. As an alternative scenario, replicated minichromosomes are randomly distributed to daughter cells during amitosis. However, only the progeny carrying correct dosages of housekeeping genes will survive, whereas copy numbers of non-housekeeping genes are allowed to drift unless they are required for a specific condition (Fig. 7). Under this scenario, the CNV pattern is shaped by natural selection and the cells do not develop specific mechanisms to adjust it. Although the second scenario sounds much more straightforward compared to the first one, it imposes a high fitness cost on the whole population. Through random segregation of minichromosomes, a certain proportion of daughter cells are likely deemed unfit since they do not have the correct composition of dosage-sensitive genes. More experiments are required to resolve this issue.

Why does *P*. *bursaria* exhibit and tolerate such a broad range of CNV? In other eukaryotic organisms, such large-scale CNV regulation can only be observed in asexual populations when cells encounter drastic environmental challenges [100, 101]. It is known that the genetic material of MACs is specific to the asexual life cycle of ciliates and that new MACs are generated during each sexual cycle. Is it possible that the high genome plasticity observed in *P*. *bursaria* represents a general and effective strategy to regulate gene dosage and expression without complex transcriptional regulatory networks? Further investigation of the ciliate genome will help us understand the general rules of how high genome plasticity is achieved and tolerated, especially during acute stress conditions.

## Conclusions

Unlike other sequenced *Paramecium* species, the macronuclear genome of *P*. *bursaria* exhibits a wide range of gene copy number variation. We analyze the patterns of CNV between different populations of *P*. *bursaria* and reveal that the CNV patterns are partially conserved. The group of genes with consistent CNV patterns among populations comprises sequence-conserved genes with housekeeping functions, whereas the group with variable CNV patterns often includes environmental response or species-specific genes. We further show that mRNA levels are partially correlated with gene copy number. Our data suggest that *P*. *bursaria* exhibits high plasticity and tolerance to gene copy number variation, which may play a general role in allowing cells to adapt to different environments.

## Methods

### Strains and culture conditions

The *P*. *bursaria* strains Dd1, KM2, HK1, and STL3 were obtained from the Symbiosis Laboratory, Yamaguchi University (http://nbrpcms.nig.ac.jp/paramecium/?lang=en), with partial support from the National Bio-Resource Project of the Japan Agency for Medical Research and Development. The endogenous endosymbiont in all of those strains is *Chlorella variabilis*. Original collection locations and dates of all strains are presented in Table S9 [see Additional file 2]. The DK1 and DK2 strains are descendants of Dd1 and KM2. To generate the progeny of Dd1 and KM2, aposymbiotic cells of Dd1 and symbiotic cells of KM2 in the early stationary phase were collected and washed twice with modified Dryl’s solution (in which KH_2_PO_4_ was used instead of NaH_2_PO_4_·2H_2_O). The cells were starved for 2 days and several hundred cells were mixed. After 5 h, each of the conjugating pairs was transferred to individual modified Dryl’s solution droplets. After the two exconjugants separated, we immediately isolated each of them to establish an independent clone. The clones were fed with *K*. *pneumoniae*-infusion lettuce medium (2.5% Boston lettuce juice in modified Dryl’s solution) after at least 2 days to avoid macronuclear regeneration. After the F1 cells started propagating, we used PCR followed by Sanger sequencing to check the genotype of specific regions in the progeny. If the genotype was a combination of both parents, then the cells were used for further analyses. The primers used for PCR are listed in Table S10 [see Additional file 2]. Both DK1 and DK2 cells showed a period of sexual immaturity after conjugation, which is consistent with the phenotype of true cross-fertilized clones. Lastly, we analyzed the whole-genome sequencing data and compared unique sequence variations (see the “Whole-genome analysis” section). Both DK1 and DK2 contained a proportion of, but not all, unique sequence variations from Dd1 and KM2 (Additional file 1: Fig. S8), providing direct evidence that DK1 and DK2 were real F1 progeny of Dd1 and KM2. The sexually reproduced strains DK1 and DK2 were generated on 25 November 2016 and 15 August 2019, respectively.

*P*. *bursaria* cells were grown in lettuce medium and fed with *Klebsiella pneumoniae* (NBRC 100048 strain). The cell cultures were kept at 23 °C with a light to dark cycle of 12 h:12 h. Fresh bacteria-containing lettuce medium was added every 2 days. The cells entered early stationary phase 1 day after their last feed.

To obtain aposymbiotic *Paramecium* strains, green symbiotic cells were treated with cycloheximide (10 μg/ml) as previously described [102].

### Genomic DNA preparation

Aposymbiotic cells of Dd1 in early stationary phase were starved for two further days and collected using a filtering apparatus with 11-μm-pore-size nylon membrane (NY1102500, Millipore, Burlington, MA, USA). To isolate macronuclei, ~ 2 × 10^6^ cells were washed twice with modified Dryl’s solution and lysed using an equal volume of 0.25 M TCMS buffer (10 mM Tris-HCl pH 8.0, 2 mM CaCl_2_, 8 mM MgCl_2_, 0.25 M sucrose) with 0.3% (w/v) NP-40. Cell extract was sonicated (~ 100 watts; Sonicator 3000, Misonix Inc., Farmingdale, NY, USA) for 6 min to dissociate the macronuclei and micronuclei. We carefully loaded 7 ml of the extract onto 8 ml of 1.6 M TCMS buffer in a 15-ml falcon tube and centrifuged at 1800 rcf and 4 °C for 15 min. The macronuclear pellet was washed once with 10 ml 0.25 M sucrose and again centrifuged at 1800 rcf and 4 °C for 15 min. The precipitate was collected and a small amount was stained with SYTOX Green to calculate the ratio of macronuclei and micronuclei. Only precipitates with a ratio of MAC number to total nucleus number > 0.8 were used. A previous study reported the ratio of DNA content of micronucleus and macronucleus in *P*. *bursaria* to be approximately 1:23 [52]. After we had enriched for MAC, reads derived from MIC were low enough to be discounted. The DNA was extracted using QIAGEN Genomic-tip 20/G (Cat No.10223, QIAGEN, Venlo, Netherlands).

To isolate total genomic DNA from whole cells, ~ 1 × 10^6^ cells were washed twice with modified Dryl’s solution and lysed by an equal volume of 0.25 M TCMS buffer with 0.3% (w/v) NP-40. Genomic DNA was extracted using QIAGEN Genomic-tip 20/G (Cat No.10223, QIAGEN).

### RNA preparation and data analysis

Approximately 1 × 10^5^ Dd1 cells in early stationary phase were collected using a filtering apparatus with 11-μm-pore-size nylon membrane and washed twice with modified Dryl’s solution. Total RNA was extracted using TRI Reagent (T9424, Sigma-Aldrich) and RNeasy Mini kit (Cat No. 74106, QIAGEN) following the manufacturer’s instructions.

RNA-seq libraries were prepared with the Illumina TruSeq Stranded mRNA LT Sample Prep Kit (Illumina, San Diego, CA, USA), and sequenced using an Illumina Nextseq platform. The reads were quality-trimmed using Trimmomatic v0.36 with options ILLUMINACLIP = 2:30:10, LEADING = 3, TRAILING = 3, SLIDINGWINDOW = 4:15, and MINLEN = 36. The transcripts per million (TPM) for each sample was quantified using Salmon v0.9.1 with option numBootstraps = 200; gcBias [103].

### Pulsed-field gel electrophoresis and southern blot

Cells of different strains and species (~ 1.5 × 10^4^ cells per plug for *P. bursaria*, ~ 5 × 10^5^ cells per plug for *Tetrahymena thermophila* BII) were washed twice in ET buffer (100 mM EDTA, 10 mM Tris buffer [pH 8.0]) and suspended in 100 μl ET buffer. The liquid was mixed with 200 μl 1.5% low gelling temperature agarose (A9414, Sigma-Aldrich, St. Louis, MO, USA) and solidified in a casting mold (Biometra, Göttingen, Germany). The agarose plugs were incubated in lysis solution (10 mM Tris buffer [pH 8.0], 1% SDS, and 1 mg/ml proteinase K, 0.1 M EDTA) overnight at 50 °C and washed for 1 h three times in TE buffer (50 mM EDTA, 10 mM Tris, pH 8.0) at room temperature. Subsequently, the agarose plugs were inserted into the well of a 1% gel (A2929, Sigma-Aldrich) to perform pulsed-field gel electrophoresis in the CHEF DR II system (Bio-Rad, Hercules, CA, USA) (0.5X TBE, ramping switch time of 2.1 s to 54.1 s, 6 V/cm, angle 120° for 17 h). The Lambda Ladder-CHEF DNA Size Standard (#170-3635, Bio-Rad) was used as a size marker. Visualization was performed after staining with ethidium bromide.

Total genomic DNA of the aposymbiotic DK1 strain was labeled with Digoxigenin (DIG) by using DIG-High Prime (Cat. No. 11585606910, Sigma-Aldrich) and the specific probes for mtDNA, the PBCV-1 genome, and gene 000334F_H1.12 (a gene encoding the homolog of alpha-tubulin) were generated using PCR DIG Probe Synthesis Kit (Cat. No. 11636090910, Sigma-Aldrich). The DNA fragments separated on the agarose gel were transferred to an Immobilon-NY+ nylon membrane (INYC00010, Millipore) and hybridized with different probes at 42 °C in the DIG Easy Hyb buffer prepared from DIG Easy Hyb granules (Cat. No. 11796895001, Sigma-Aldrich). After overnight hybridization, the membrane was washed twice with 2X SSC and 0.1% SDS at 68 °C for 15 min and then washed twice with 0.5X SSC and 0.1% SDS at 68 °C for 15 min. For immunological detection, the membrane was incubated in 2X blocking buffer (2% Casein, 0.1 M Maleic acid, 0.15 M NaCl, pH 7.5) at 37 °C for 30 min and incubated with anti-DIG-AP, Fab fragment (1:10000, 11093274910, Roche Applied Science, Penzberg, Germany) at 37 °C for 30 min. The membrane was washed twice with washing buffer (0.1 M maleic acid, 0.15 M NaCl, 0.3% Tween 20, pH 7.5) at 37 °C for 15 min and equilibrated in detection buffer (0.1 M Tris, 0.1 M NaCl, 0.05 M MgCl_2_, pH 9.5) at 37 °C for 5 min. The hybridization fragments were detected using CSPD-ready to use (Cat. No. 11755633001, Sigma-Aldrich) according to the manufacturer’s instructions.

### Genome sequencing and assembly

A 20-kb insertion genomic DNA library was prepared according to a standard protocol and sequenced using the PacBio Sequel platform (Pacific Biosciences, Menlo Park, CA, USA) of the NGS core of Academia Sinica (http://ngs.biodiv.tw/NGSCore/) to obtain long reads data. Paired-end libraries were prepared by a standard protocol and sequenced using the Illumina Miseq and Nextseq platforms of the Genomics core of IMB (http://www.imb.sinica.edu.tw/mdarray/). The reads were quality-trimmed using Trimmomatic v0.36 with options ILLUMINACLIP = 2:30:10, LEADING = 3, TRAILING = 3, SLIDINGWINDOW = 4:15, and MINLEN = 36. The subreads were assembled using Falcon v.0.4.0 with options overlap minlen = 1000, to graph minlen = 1000, max_diff = 100, max_cov = 100 and min_cov = 5 [104], and the sequence was polished by Illumina reads using Pilon v1.22 [105].

We assembled 614 contigs and removed 17 contigs derived from bacterial contamination judging from their GC content and similarity to bacterial sequences according to the NCBI database. Many diploid genomes contain regions with high levels of heterozygosity, which increases the difficulty of distinguishing two haplotypes [106, 107]. If the regions with highly divergent alleles are assigned as two alternate contigs, the assembled genome will become fragmented and bigger than the real genome. We used Redundans v0.11 [108] to generate the haploid genome. The default criteria for Redundans (established from simulation and real data by the developers) to determine alternative contigs as haplotypes are as follows. First, the alignment length should be more than 80% of the shorter contig. Second, the sequence identity should be greater than 50%. To increase stringency in our analysis, we used 70% as a cutoff for the second criterion. We used MUMMER v3.23 [109] to generate the haploid genome and manually joined the overlapping contigs. To distinguish the two haplotypes, we identified variations by GATK v4.0.3 [110] with default settings and filtered out the variants located in the regions with lower depths (≤ 30) or had a lower variant read depth (≤ 10). Among the 661,718 heterozygous sites identified between H1 and H2 haplotypes of Dd1, only 40 sites have more than two genotypes. The results suggested that the majority of our assembly did not collapse duplication regions into a single locus. We constructed the diploid genome by HAPCUT2 v1.0 [111] according to the variant information acquired from the GATK algorithm. The effect of variants was examined by SNPEFF 4.4 [112]. The accession numbers of each sequencing dataset can be found in Table S9 (see Additional file 2). The mapping rates for the reads from RNA-seq and DNA-seq were 96.4% and 95.6%, respectively, suggesting that our assembly represents most of the functional genome.

### Genome annotation

Gene annotation was performed by comparing our *P*. *bursaria* RNA-seq data generated from the Dd1 strain and protein sequences from *P. caudatum* and *P. tetraurelia* acquired from ParameciumDB (http://paramecium.i2bc.paris-saclay.fr) using the pipeline described in Arnaiz et al. [113]. We annotated the genes for both haplotypes and then used the following criteria to select for the functional gene set of *P*. *bursaria*. First, we aligned gene sequences by BWA-MEM [114, 115] and grouped alleles from the same locus in the four different sequenced *P*. *bursaria* strains. If the alleles shared the same sequence positions for the start and stop sites when aligned, they were classified into a CDS subgroup (Fig. 1b). For each locus, we chose the subgroup with the highest number of alleles to identify the functional allele. If the difference between the allele numbers of the top two subgroups was equal to or less than one, we chose the subgroup with a longer coding region. Once the subgroup was selected, we calculated alignment bit scores for all allele pairs using BLASTp and selected the allele with the highest average score as the representative functional gene. Potential protein domains of each annotated gene were identified using InterProscan 5.30–69.0 [116], and the gene was assigned to gene ontology terms according to its protein domains.

To assess the completeness of our genome assembly, we used the following criteria to filter the alignment of Core Eukaryotic Gene (CEG) sequences and our genome from BLASTP [56]. First, the alignment length had to cover over 70% of the aligned CEG sequence. Second, the alignment *E*-value should be less than 1e−10 [36].

### Calculation of nucleotide diversity π of intergenic regions

We used CDS subgroups containing more than four alleles in the populations (*n* = 12,435) to calculate the nucleotide diversity per site. We investigated the region upstream of the start codon of each gene until the point where it encountered the next gene, but with a maximum length of 500 bp. Nucleotide diversity was calculated using the formula π = 2*pqn*/(*n* − 1), where *p* and *q* are the major and minor allele frequencies and *n* is the total number of alleles in each group [117].

### Construction of species phylogenetic tree

We identified homologs among different ciliate species using OrthoFinder v2.0.0 [61] with default settings. In each orthogroup, an individual species may contribute more than one ortholog if there is a recent gene or genome duplication event in that species. For example, *P*. *tetraurelia* often contributes multiple genes to each orthogroup since it has experienced two whole-genome duplication events. Some orthogroups may contain genes from only one species if they are species-specific genes. To draw the Venn diagram in Fig. 2, different types of orthogroups (e.g., the orthogroup containing orthologs from all species) were collected and the total gene numbers from each species were counted. To construct the phylogenetic tree, we chose genes having only one ortholog in each species (*n* = 493) to perform the analysis. The protein sequences of each orthogroup were aligned independently using MUSCLE v3.8.31 [118]. The conserved blocks from the multiple sequence alignment of each orthogroup were selected using Gblocks v0.91b [119] and concatenated. IQ-TREE v1.6.10 was used to select the best-fit model for sequence evolution (LG+F+I+G4) and to reconstruct the phylogenetic tree according to a Maximum Likelihood approach [120, 121]. The graph was drawn using the ETE toolkit [122].

### Gene ontology enrichment

GO categories with fewer than three genes were excluded. Enrichment scores were calculated by dividing the proportion of the genes of interest classified into the indicated category by the proportion of those genes in the genomic background. The significance of enrichment was analyzed using a hypergeometric test (phyper module) in R, and *p* values were adjusted using the Benjamini-Hochberg method [123].

### Identification of protein family and gene lineage expansions

The protein domains of each gene of *P*. *bursaria*, *P*. *caudatum*, and *P*. *tetraurelia* were identified using InterProscan 5.30–69.0 [116]. Each protein family contains both duplicated genes (which share high levels of sequence similarity) and non-duplicated genes that carry the same protein domain. To investigate the expanded protein families specific to *P*. *bursaria*, we calculated the gene number of each protein family in each species. A two-proportional test was used to test significance in three species, with a *p* value threshold equal to or smaller than 0.05 after Benjamini-Hochberg adjustment. To examine the orthologs specifically duplicated in *P*. *bursaria*, we categorized the genes in *P*. *bursaria*, *P*. *caudatum*, and *P*. *tetraurelia* into orthogroups depending on the protein similarity using OrthoFinder v2.0.0 [61] with default settings. A two-proportional test was used to test the orthogroups with a significantly different gene number in the three species, with a *p* value threshold equal to or less than 0.05 after Benjamini-Hochberg adjustment.

### Whole-genome analysis

For copy number variation (CNV) analysis, trimmed reads were mapped via BWA-MEM and read depth was analyzed using the SAMtools program [124]. The copy number of each contig was calculated by normalizing contig length and read depth to the whole genome coverage derived from the SAMtools bedcov module. The copy number of each gene was also calculated by normalizing gene length and read depth to the whole genome coverage derived from the SAMtools bedcov module. The coefficient of variation (CV) for gene copy number was calculated using the copy numbers of each gene in all four strains.

We used a homemade Perl script to identify the Illumina reads that contained telomeric repeats. For Illumina data, the reads needed to contain at least 18 bp of telomere repeats with an allowance for one mismatch. For PacBio data, the reads needed to contain at least 30 bp of telomere repeats with an allowance of five mismatches. We used the same method described above for CNV analysis to calculate the read counts in 2-kb windows for both Illumina and PacBio reads (Additional file 4: Table S6). For the correlation analysis of coverage between two sets of data, the read depth in 2-kb windows was normalized to the window length to acquire the coverage, and Spearman’s rank correlation was used to examine the correlation between two sets of data.

To identify the unique sequence variants of each strain, we identified variations by GATK v4.0.3 [110] and used the H1 haplotype of the Dd1 strain as the reference genome. The BEDTools was used to calculate the shared sequence variations between strains [125]. To calculate the divergence between Dd1 and other strains, the sequence of a diploid strain X was first compared with the reference genome (Dd1-H1). If a difference was detected, it would then be compared to another haplotype (Dd1-H2) of Dd1. Only if it differed from both Dd1-H1 and Dd1-H2, it was counted as a unique SNP (Additional file 2: Table S2). Such SNPs could be homozygous or heterozygous in the strain X, but it would be counted only once. The total SNP number was divided by the reference genome size to get the divergence (Additional file 2: Table S2). For identification of alleles derived from each parent in the DK1 and DK2 strains, we compared Dd1-H2, KM2, DK1, or DK2 sequences to the reference genome (Dd1-H1) and identified the variants. We then counted the variant numbers shared between different strains to draw the Venn diagram (Additional file 1: Fig. S8).

To calculate the evolutionary rates of individual genes, pairwise Ka/Ks was calculated for all alleles in the functional allele subgroup (Fig. 1b), and then all Ka/Ks values were averaged for each gene. Only the CDS subgroups containing more than three alleles were used in this analysis. The pairwise Ka/Ks value was calculated in PAML 4.9e [126].

### Quantitative PCR

The genomic DNA was diluted to an appropriate concentration and then subjected to quantitative PCR using gene-specific primers (Additional file 2: Table S10) and Fast SYBR Green master mix in an Applied Biosystems 7500 Fast Real-Time PCR System (Applied Biosystems, Waltham, MA, USA). Data were analyzed using the built-in analysis program.

### Clustering analysis and the bootstrap method

We used the clusterdist function in ClusterScan v.0.2.2 [127] to calculate the number of genes that form clusters in the genome for the conserved group with the option dist = 500. We counted the number of conserved CN genes that were defined inside the clusters and used to calculate their proportion in the conserved CN group. To generate the reference genome background set, we randomly picked the same number of conserved CN genes from the whole genome 1000 times and calculated the number of genes in the clusters. After we acquired the distribution of clustering percentage from the random sampling datasets, we tested whether the observed clustering percentage of the conserved CN group was significantly deviated from the random distribution.

### Motif discovery

The aligned reads containing telomeric repeats on the left or right end with respect to the contig were mapped separately to the reference genome using BWA-MEM. We calculated the read depth of telomere-containing reads in a 0.5-kb interval using the SAMtools bedcov module. DNA sequences of the intervals having at least four telomere-containing reads were extracted and subjected to motif analysis after removing the telomeric sequences. Motif discovery was performed using MEME and the occurrence of motifs in the input regions was searched by FIMO in the MEME suite v5.0.5 [128].

## Supplementary Information

**Additional file 1: Figure S1.** De novo assembly of the *P*. *bursaria* MAC genome. **Figure S2.** *P*. *bursaria* introns have very conserved 5′ and 3′ splice sites. **Figure S3.** PFG Southern blot analysis of the genomic DNA of different *P*. *bursaria* strains. **Figure S4.** The extensive chromosome breakage pattern in the MAC. **Figure S5.** A model showing how highly variable breaking sites lead to non-uniform gene dosage. **Figure S6.** Contig copy number is uniform in *T*. *thermophila*. Different *P*. *bursaria* strains show similar patterns of copy number distribution. **Figure S7.** Conserved GA-rich motifs are found near chromosome breakage sites. **Figure S8.** DK1 and DK2 strains are the real F1 progeny of Dd1 and KM2.

**Additional file 2: Table S1.** Basic statistics for the coding regions of the two haplotypes of the Dd1 reference genome. **Table S2.** Statistics of sequence diversity for different *P*. *bursaria* strains compared to the H1 haplotype of Dd1. **Table S3.** Basic statistics for the intergenic and intron regions on two haplotypes of the reference genome. **Table S5.** Enriched GO terms for *P*. *bursaria* specific genes (*p*-value ≤0.05 after the Benjamini-Hochberg correction). **Table S7.** The correlation matrix (Spearman’s correlation coefficient ρ) of the gene copy number between genetically divergent strains. **Table S8.** Enriched GO terms for the conserved and non-conserved groups of genes (*p*-value ≤0.05 after the Benjamini-Hochberg correction). **Table S9.** DNA and RNA sequencing library list and data accession numbers. **Table S10.** Primer list used in qPCR and Southern blot and F1 progeny validation.

**Additional file 3: Table S4.** Splicing-related genes identified in the budding yeast *Saccharomyces cerevisiae*.

**Additional file 4: Table S6.** Read counts of Illumina and Pacbio reads with telomeric sequences at their ends.

## Data Availability

The datasets generated and analyzed during the current study are included in this published article and its supplementary information files and available in NCBI under the accession number BioProject PRJNA556774 (https://www.ncbi.nlm.nih.gov/bioproject/PRJNA556774) and PRJNA555640 (https://www.ncbi.nlm.nih.gov/bioproject/PRJNA555640).

## Abbreviations

CNV: Copy number variation
SNP: Single nucleotide polymorphism
MIC: Micronucleus
MAC: Macronucleus
IES: Internal eliminated sequence
NHEJ: Non-homologous end joining
CDS: Coding sequence
ISV: Internal structural variation region
CEG: Core eukaryotic gene
GO: Gene ontology
qPCR: Quantitative PCR
PBCV-1: *Paramecium bursaria Chlorella* virus-1
