# Supplementary material for: Genome plasticity in Paramecium bursaria revealed by population genomics
Source: BMC Biol. 2020 Nov 30;18:180. doi: 10.1186/s12915-020-00912-2 (PMC7702705; doi:10.1186/s12915-020-00912-2)
Supplement: Supplementary file 1 — Additional file 1: Figure S1. De novo assembly of the P. bursaria MAC genome. Figure S2. P. bursaria introns have very conserved 5′ and 3′ splice sites. Figure S3. PFG Southern blot analysis of the genomic DNA of different P. bursaria strains. Figure S4. The extensive chromosome breakage pattern in the MAC. Figure S5. A model showing how highly variable breaking sites lead to non-uniform gene dosage. Figure S6. Contig copy number is uniform in T. thermophila. Different P. bursaria strains show similar patterns of copy number distribution. Figure S7. Conserved GA-rich motifs are found near chromosome breakage sites. Figure S8. DK1 and DK2 strains are the real F1 progeny of Dd1 and KM2. [file 12915_2020_912_MOESM1_ESM.pdf]

Fig. S1

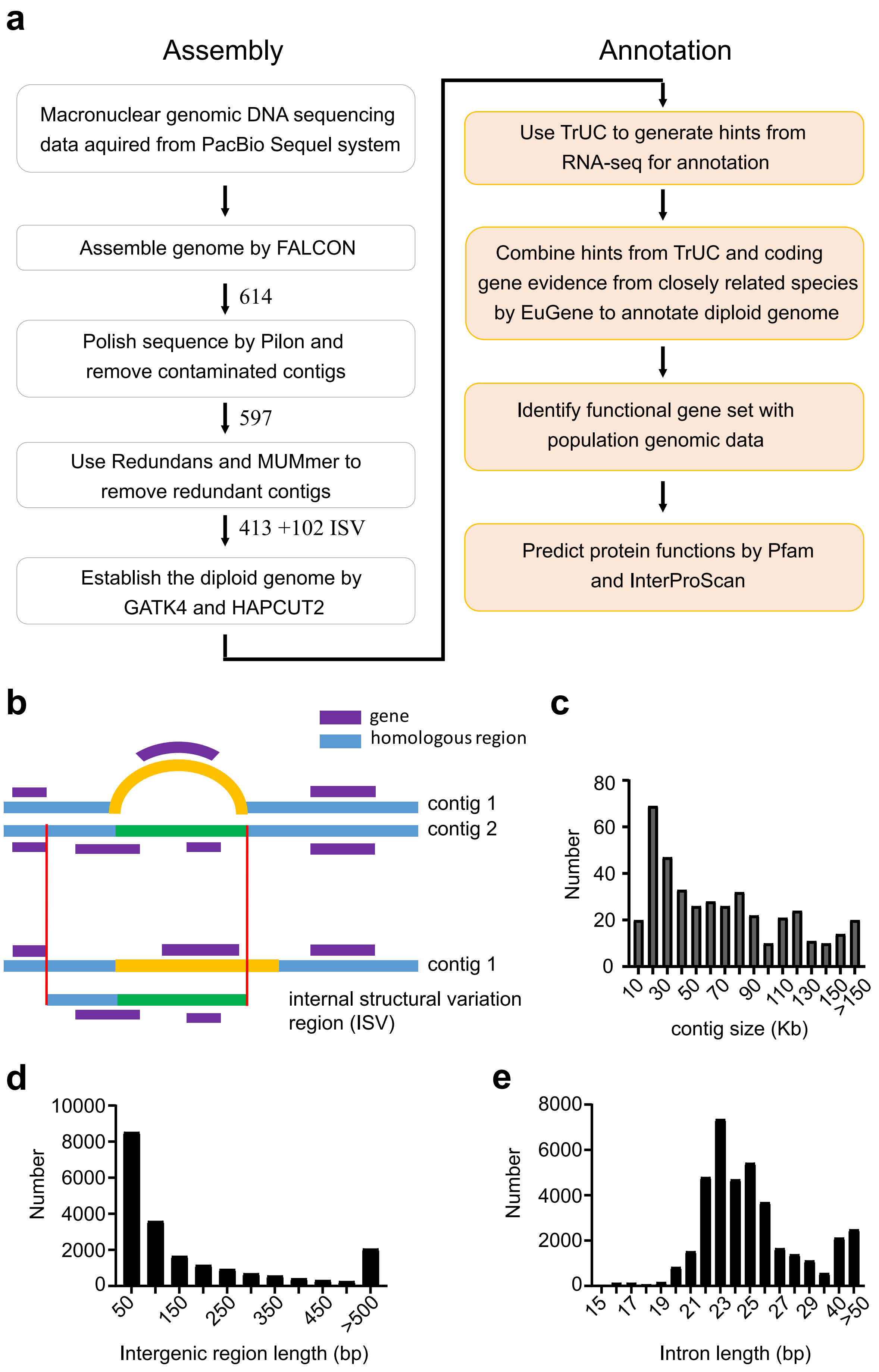
a
Assembly
Annotation
Macronuclear genomic DNA sequencing data aquired from PacBio Sequel system
Assemble genome by FALCON
614
Polish sequence by Pilon and remove contaminated contigs
597
Use Redundans and MUMmer to remove redundant contigs
413 +102 ISV
Establish the diploid genome by GATK4 and HAPCUT2
Use TrUC to generate hints from RNA-seq for annotation
Combine hints from TrUC and coding gene evidence from closely related species by EuGene to annotate diploid genome
Identify functional gene set with population genomic data
Predict protein functions by Pfam and InterProScan
b
gene
homologous region
contig 1
contig 2
contig 1
internal structural variation region (ISV)
c
Number
contig size (Kb)
d
Number
Intergenic region length (bp)
e
Number
Intron length (bp)

Fig. S2

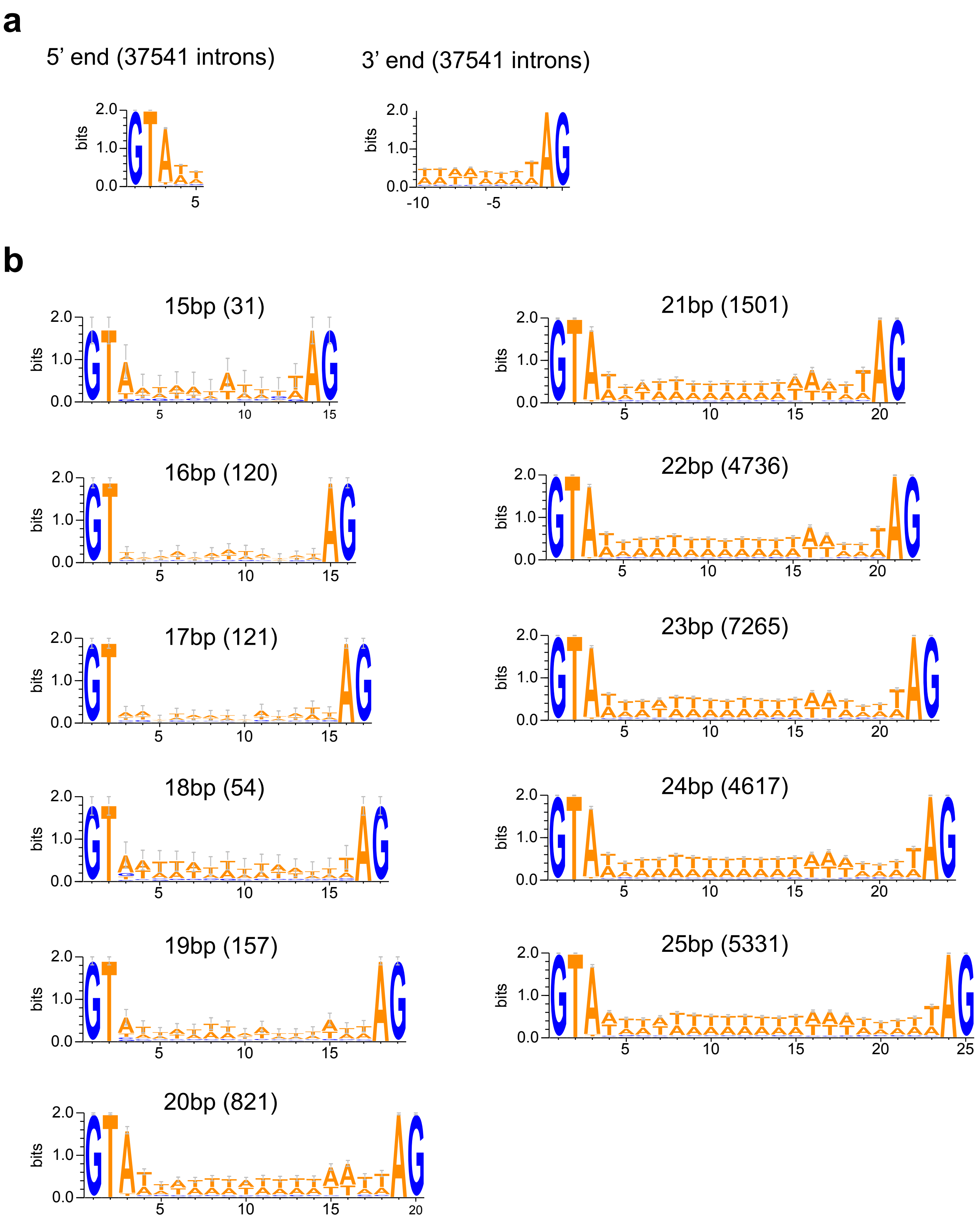
a
5’ end (37541 introns)
3’ end (37541 introns)
bits
b
15bp (31)
16bp (120)
17bp (121)
18bp (54)
19bp (157)
20bp (821)
21bp (1501)
22bp (4736)
23bp (7265)
24bp (4617)
25bp (5331)

Fig. S3

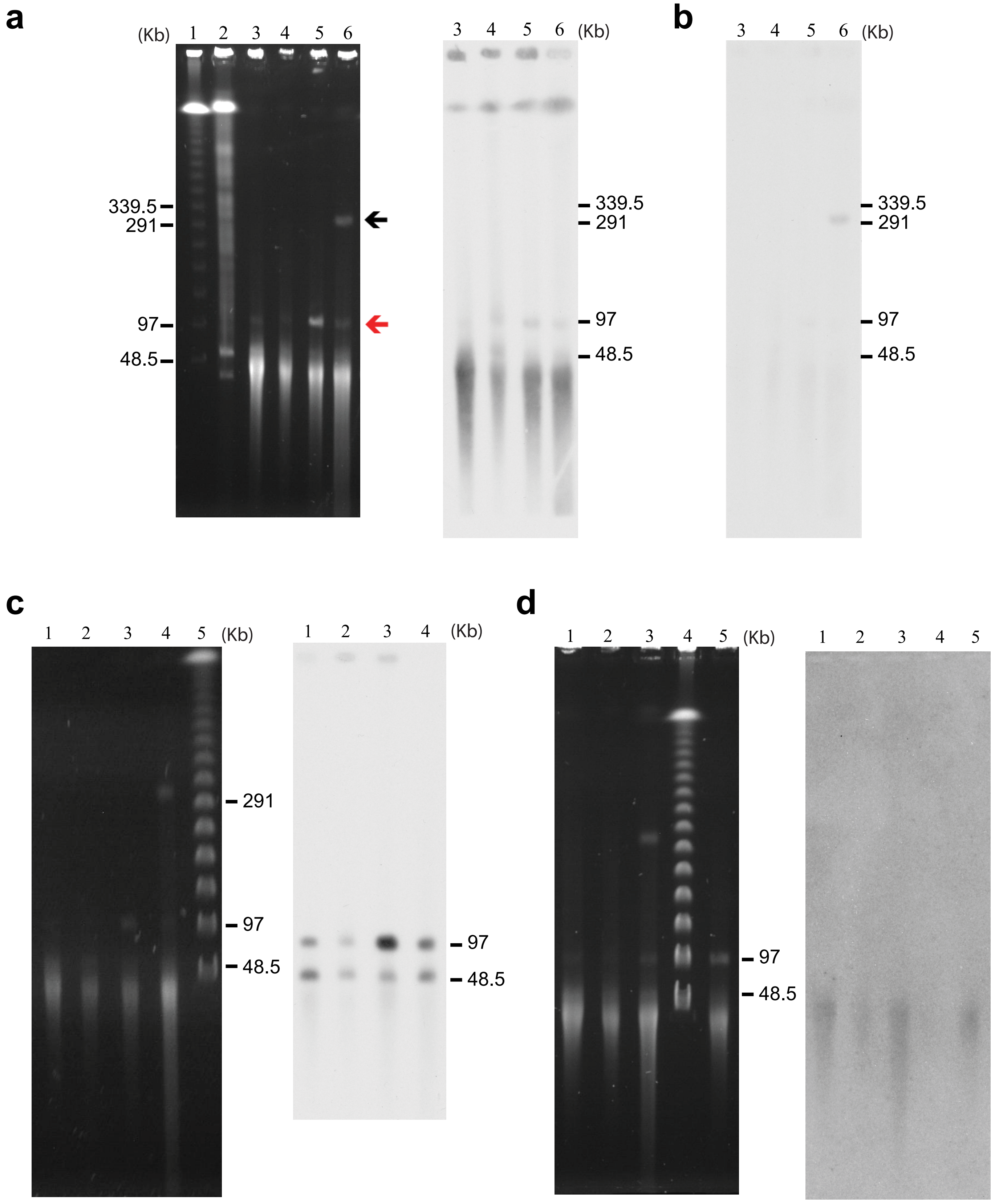
a
(Kb) 1 2 3 4 5 6
339.5
291
97
48.5
3 4 5 6 (Kb)
339.5
291
97
48.5
b
3 4 5 6 (Kb)
339.5
291
97
48.5
c
1 2 3 4 5 (Kb)
291
97
48.5
1 2 3 4 (Kb)
97
48.5
d
1 2 3 4 5 (Kb)
97
48.5
1 2 3 4 5

Fig. S4

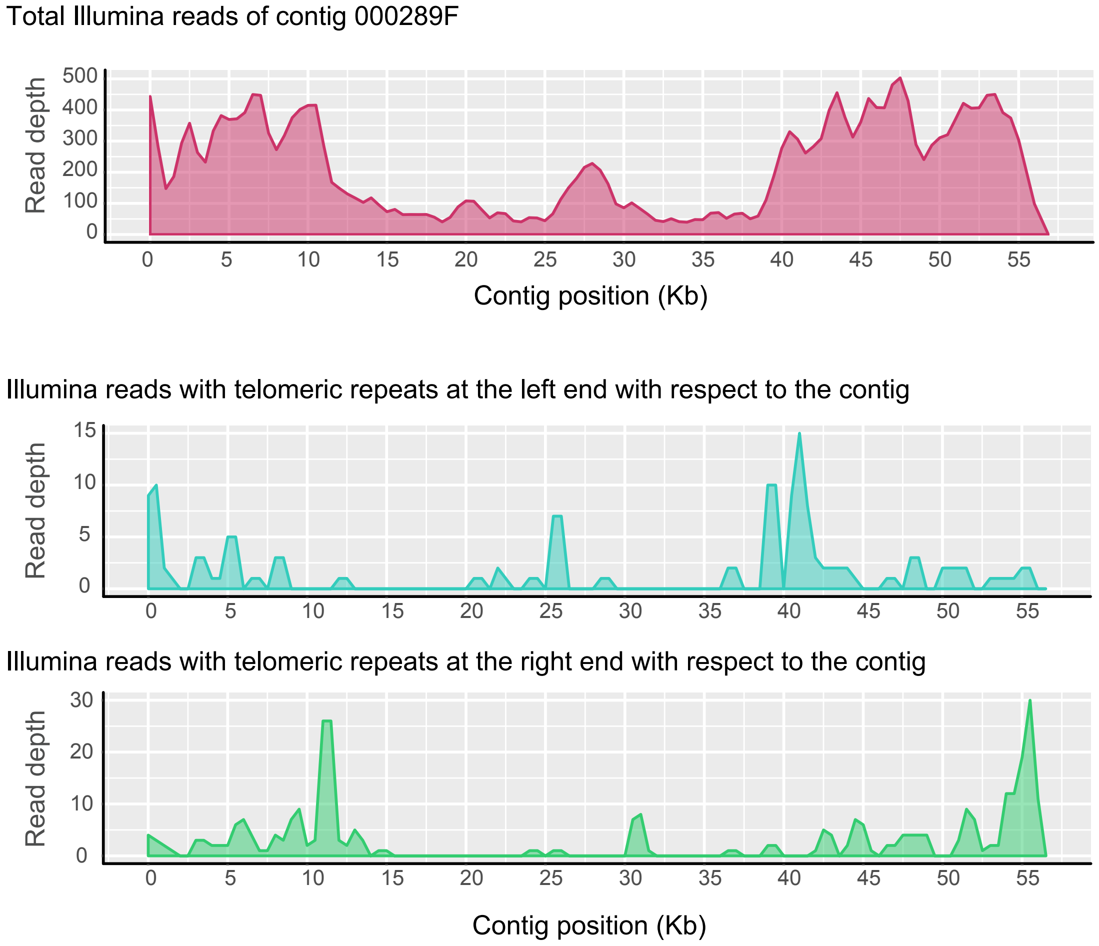
Total Illumina reads of contig 000289F
Read depth
500
400
300
200
100
0
0 5 10 15 20 25 30 35 40 45 50 55
Contig position (Kb)
Illumina reads with telomeric repeats at the left end with respect to the contig
Read depth
15
10
5
0
0 5 10 15 20 25 30 35 40 45 50 55
Illumina reads with telomeric repeats at the right end with respect to the contig
Read depth
30
20
10
0
0 5 10 15 20 25 30 35 40 45 50 55
Contig position (Kb)

Fig. S5

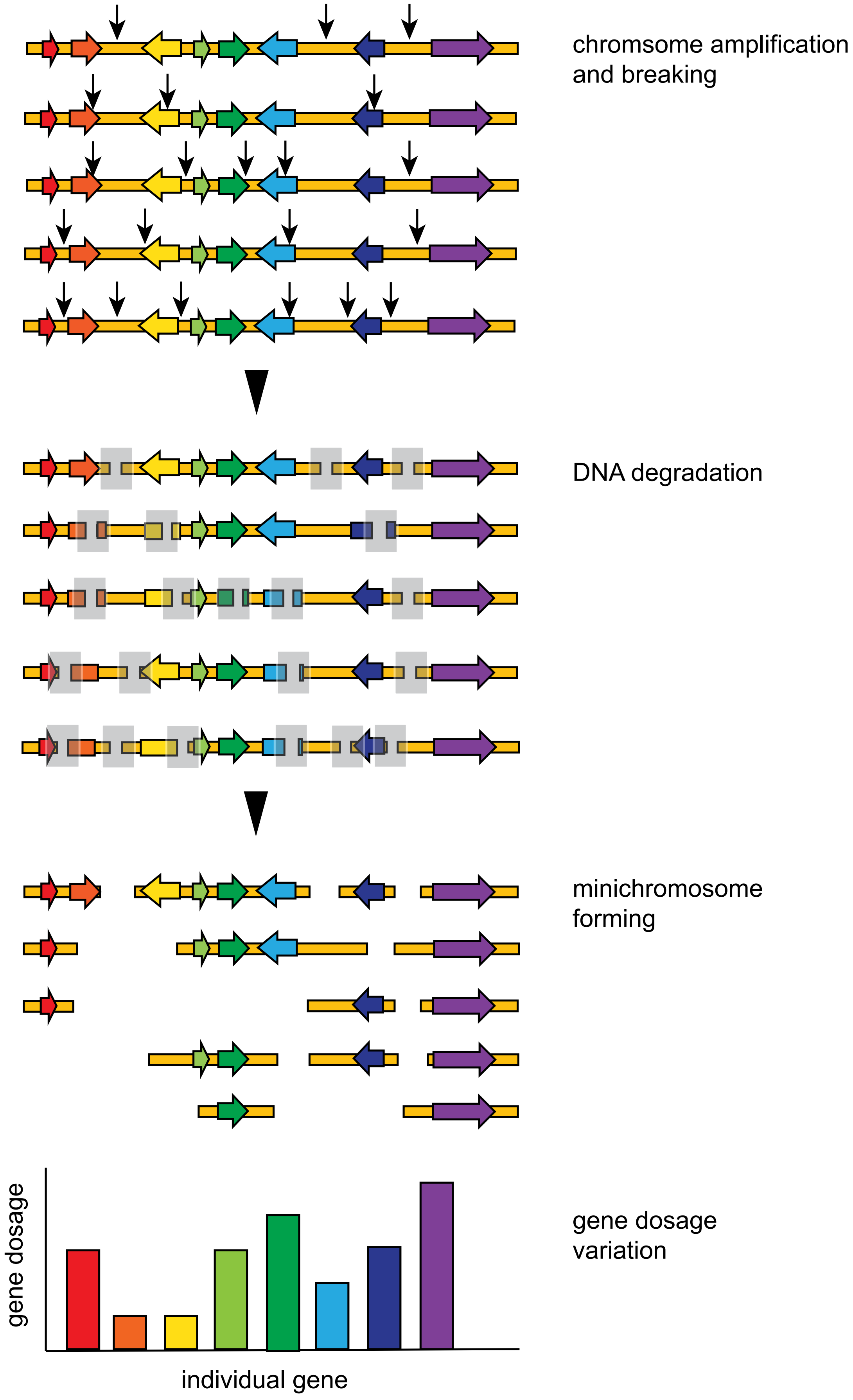
chromsome amplification
and breaking
DNA degradation
minichromosome
forming
gene dosage
variation
gene dosage
individual gene

Fig. S6

a

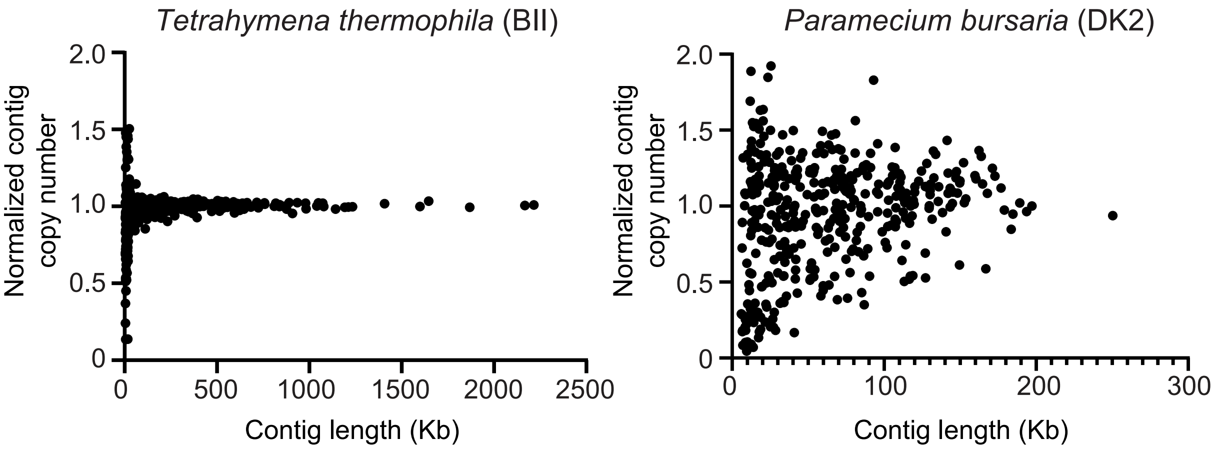
Tetrahymena thermophila (BII)
Paramecium bursaria (DK2)
Normalized contig copy number
Contig length (Kb)
2.0
1.5
1.0
0.5
0
500
1000
1500
2000
2500
100
200
300

b

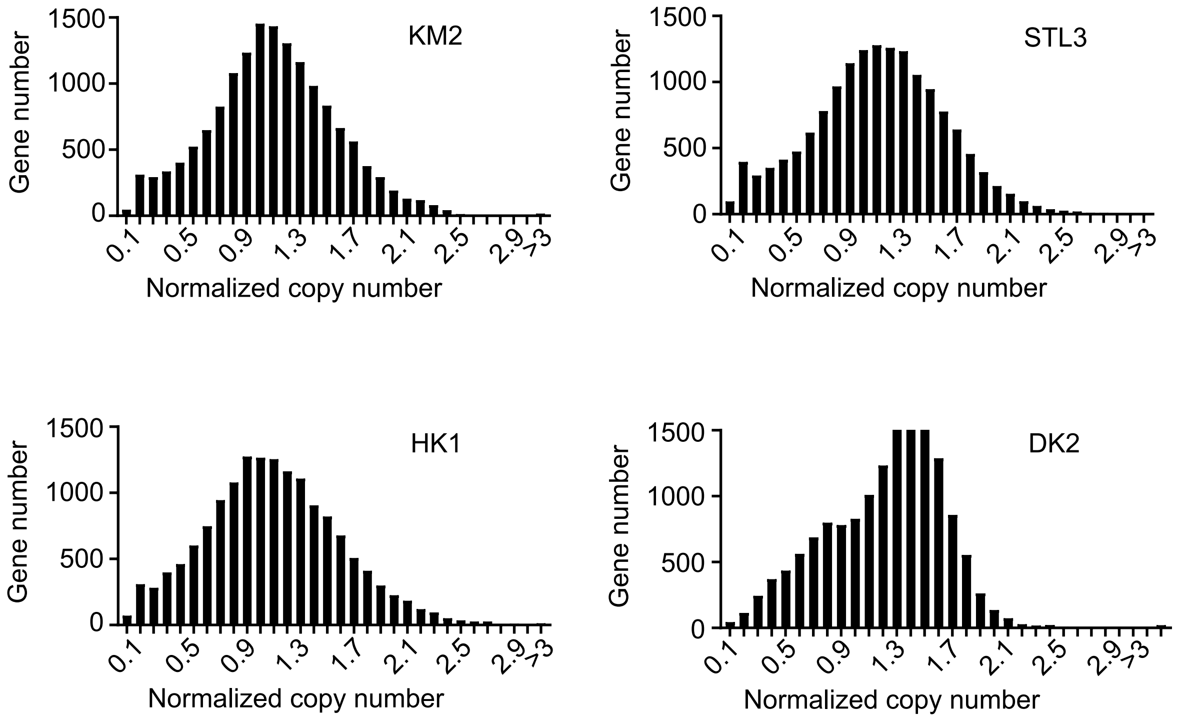
KM2
STL3
HK1
DK2
Gene number
Normalized copy number
1500
1000
500
0
0.1
0.5
0.9
1.3
1.7
2.1
2.5
2.9
>3

Fig. S7

Motif found in the regions with telomeric repeats on the left end
(E-value = 3.4e-026)
FIMO: 505/1083

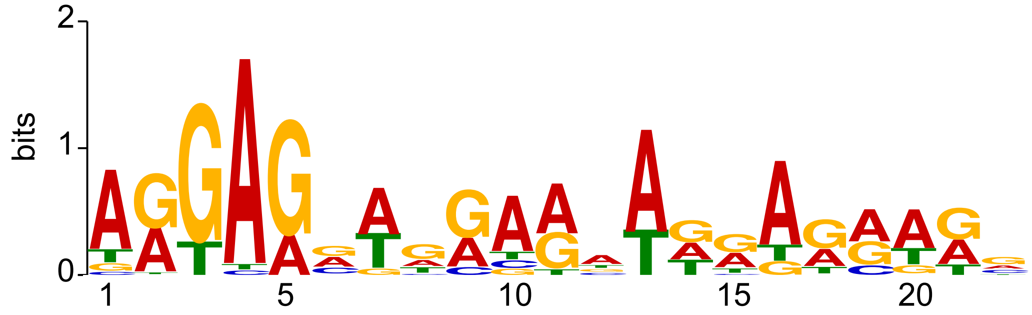

Motif found in the regions with telomeric repeats on the right end
(E-value = 8.5e-017)
FIMO: 337/1174

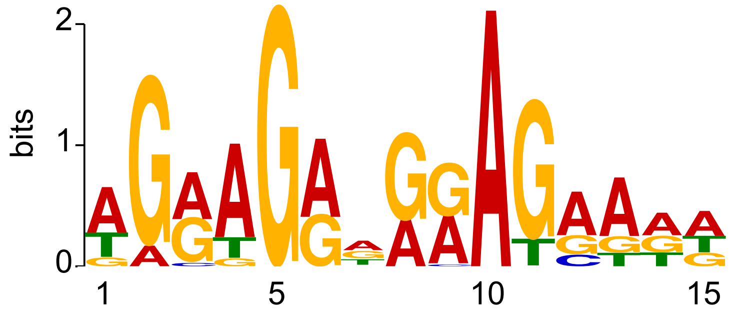

Fig. S8

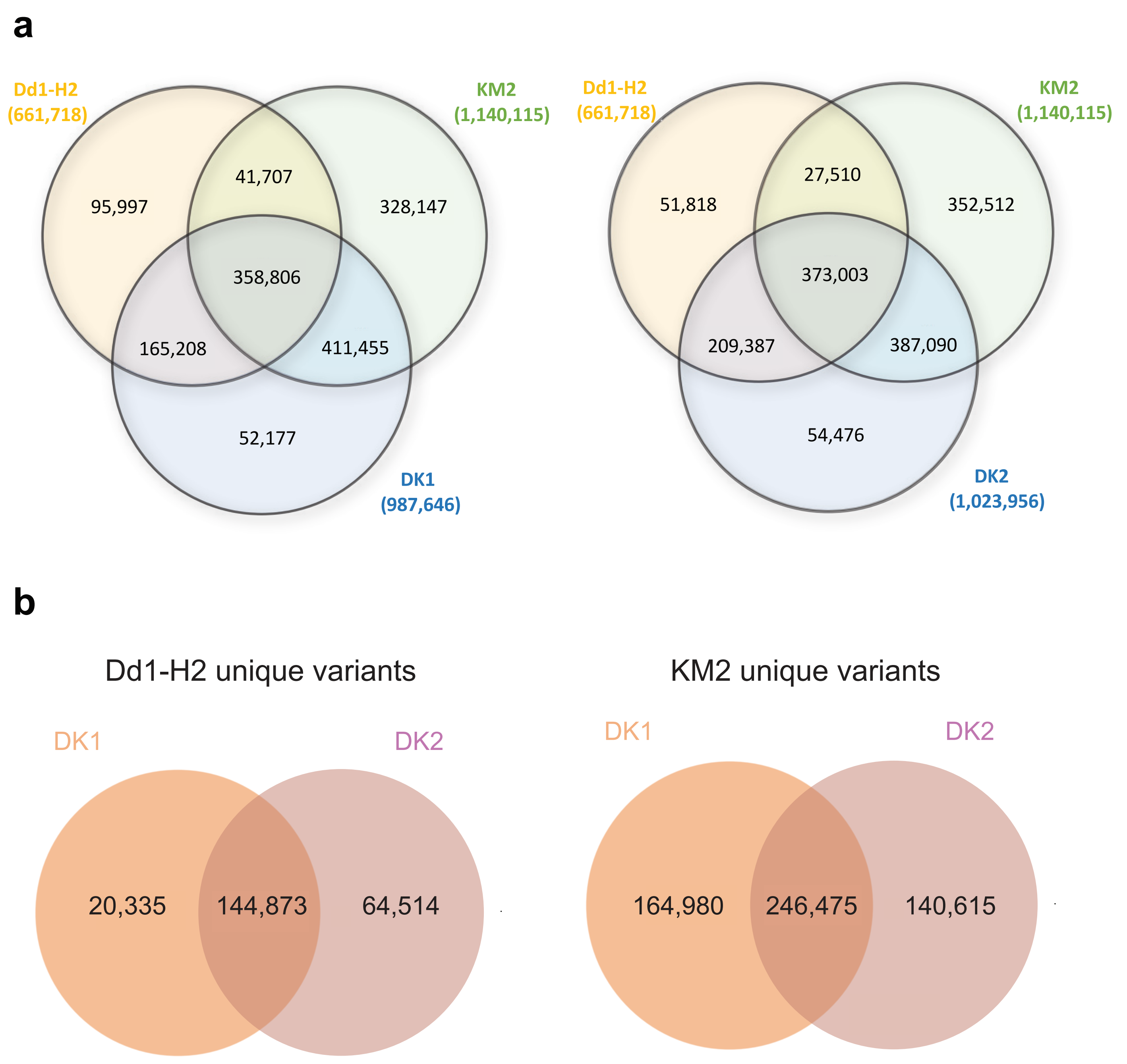
a
Dd1-H2
(661,718)
KM2
(1,140,115)
DK1
(987,646)
95,997
41,707
328,147
358,806
165,208
411,455
52,177
Dd1-H2
(661,718)
KM2
(1,140,115)
DK2
(1,023,956)
51,818
27,510
352,512
373,003
209,387
387,090
54,476
b
Dd1-H2 unique variants
DK1
DK2
20,335
144,873
64,514
KM2 unique variants
DK1
DK2
164,980
246,475
140,615
