# Supplementary material for: Genome plasticity in Paramecium bursaria revealed by population genomics
Source: BMC Biol. 2020 Nov 30;18:180. doi: 10.1186/s12915-020-00912-2 (PMC7702705; doi:10.1186/s12915-020-00912-2)
Supplement: Supplementary file 2 — Additional file 2: Table S1. Basic statistics for the coding regions of the two haplotypes of the Dd1 reference genome. Table S2. Statistics of sequence diversity for different P. bursaria strains compared to the H1 haplotype of Dd1. Table S3. Basic statistics for the intergenic and intron regions on two haplotypes of the reference genome. Table S5. Enriched GO terms for P. bursaria specific genes (p-value ≤0.05 after the Benjamini-Hochberg correction). Table S7. The correlation matrix (Spearman’s correlation coefficient ρ) of the gene copy number between genetically divergent strains. Table S8. Enriched GO terms for the conserved and non-conserved groups of genes (p-value ≤0.05 after the Benjamini-Hochberg correction). Table S9. DNA and RNA sequencing library list and data accession numbers. Table S10. Primer list used in qPCR and Southern blot and F1 progeny validation. [file 12915_2020_912_MOESM2_ESM.docx]

Table S1. Basic statistics for the coding regions on two haplotypes of the Dd1 reference genome.

| **Haplotype** | **Gene number** | **Total length (bp)** | **Avg. length (bp)** | **Min length (bp)** | **Max length (bp)** | **Median length (bp)** | **GC content (%)** |
| --- | --- | --- | --- | --- | --- | --- | --- |
| H1 | 15591 | 22411955 | 1437.49 | 106 | 23989 | 1049 | 29.7 |
| H2 | 15601 | 22401553 | 1435.9 | 104 | 20827 | 1047 | 29.7 |

Table S2. Statistics of sequence diversity of different *P. bursaria* strains compared to the H1 haplotype of the Dd1 strain.

| **Strain** | **Percentage of ref genome covered (≥ 10)** | **Mean depth of coverage (of covered genome)** | **Overlapped variants^a^** | **No. of unique SNPs^b^** | **No. of unique Indels^c^** | **Divergence (%)^d^** |
| --- | --- | --- | --- | --- | --- | --- |
| KM2 | 96.1 | 235 | 429596 | 662178 | 50605 | 2.66 |
| HK1 | 93.5 | 121 | 396607 | 532413 | 38876 | 2.13 |
| STL3 | 92.3 | 167 | 364820 | 996183 | 80926 | 4.02 |

a. Overlapped variants are heterozygous sites observed in the strain and also in Dd1. They are not included when calculating the strain divergence.

b. Unique SNPs are the variants not observed in Dd1-H1 and H2. Such SNPs could be homozygous or heterozygous, but they would be counted only once.

c. Unique indels are the insertions/deletions not observed in Dd1-H1 and H2. Each site is counted only once.

d. The total numbers of unique SNPs and indels are divided by the reference genome size (Dd1-H1) to get the divergence.

Table S3. Basic statistics for the intergenic and intron regions on two haplotypes of the reference genome.

| **Region** | **Total number** | **Total length (bp)** | **Avg. length (bp)** | **Min length (bp)** | **Max length (bp)** | **Median length (bp)** | **GC content (%)** |
| --- | --- | --- | --- | --- | --- | --- | --- |
| H1_intergenic | 15109 | 2958135 | 195.79 | 1 | 14037 | 44 | 22.4 |
| H2_intergenic | 15119 | 2998777 | 198.35 | 1 | 14042 | 44 | 22.4 |
| H1_intron | 37655 | 1035950 | 27.51 | 15 | 101 | 24 | 17 |
| H2_intron | 37601 | 1034066 | 27.50 | 15 | 100 | 24 | 17 |

The intergenic regions from the contig end to the first gene were excluded.

Table S5. Enriched GO terms for *P. bursaria* specific genes (*p*-value ≤ 0.05 after the Benjamini-Hochberg correction).

| GO term | No. of genes | Enrichment score | *p*-value | Category | Description of GO term |
| --- | --- | --- | --- | --- | --- |
| GO:0006468 | 121 | 4.80 | 1.33E-02 | BP | protein phosphorylation |
| GO:0006855 | 5 | 4.46 | 1.68E-02 | BP | drug transmembrane transport |
| GO:0016192 | 24 | 2.81 | 9.85E-05 | BP | vesicle-mediated transport |
| GO:0000160 | 18 | 2.36 | 1.11E-03 | BP | phosphorelay signal transduction system |
| GO:0007166 | 3 | 1.83 | 1.35E-02 | BP | cell surface receptor signaling pathway |
| GO:0006886 | 22 | 1.68 | 2.93E-05 | BP | intracellular protein transport |
| GO:0007165 | 17 | 1.26 | 2.51E-02 | BP | signal transduction |

GO categories are shown only if they include at least three genes. Enrichment scores were calculated by dividing the proportion of target gene set classified into the indicated category by the proportion of those in the genome background. BP: Biological process.

Table S7. The correlation matrix (Spearman correlation coefficient ρ) of the gene copy number between genetically divergent strains.

| Strain | Dd1 replicate 1 | Dd1 replicate 2 | HK1 replicate 1 | HK1 replicate 2 | STL3 replicate 1 | STL3 replicate 2 | Km2 replicate 1 | Km2 replicate 2 |
| --- | --- | --- | --- | --- | --- | --- | --- | --- |
| Dd1 replicate 1 | 1.00 |  |  |  |  |  |  |  |
| Dd1 replicate 2 | 0.91 | 1.00 |  |  |  |  |  |  |
| HK1 replicate 1 | 0.63 | 0.69 | 1.00 |  |  |  |  |  |
| HK1 replicate 2 | 0.53 | 0.49 | 0.80 | 1.00 |  |  |  |  |
| STL3 replicate 1 | 0.61 | 0.65 | 0.65 | 0.46 | 1.00 |  |  |  |
| STL3 replicate 2 | 0.49 | 0.44 | 0.40 | 0.47 | 0.78 | 1.00 |  |  |
| Km2 replicate 1 | 0.63 | 0.63 | 0.59 | 0.49 | 0.59 | 0.48 | 1.00 |  |
| Km2 replicate 2 | 0.45 | 0.40 | 0.38 | 0.49 | 0.39 | 0.51 | 0.79 | 1.00 |

The smaller size of genes contributes to higher noise in the calculation of copy number, leading to reduced overall coefficient correlations.

Table S8. Enriched GO terms for the conserved and non-conserved groups of genes (*p*-value ≤ 0.05 after the Benjamini-Hochberg correction).

| GO term | No. of genes | Enrichment score | *p*-value | Category | Description of GO term |
| --- | --- | --- | --- | --- | --- |
| Conserved group |  |  |  |  |  |
| GO:0051016 | 3 | 3.45 | 0.00E+00 | BP | barbed-end actin filament capping |
| GO:0006370 | 3 | 3.45 | 0.00E+00 | BP | 7-methylguanosine mRNA capping |
| GO:0019441 | 3 | 3.45 | 0.00E+00 | BP | tryptophan catabolic process to kynurenine |
| GO:0019236 | 3 | 3.45 | 0.00E+00 | BP | response to pheromone |
| GO:0003341 | 5 | 2.47 | 1.09E-02 | BP | cilium movement |
| GO:0016310 | 15 | 1.85 | 5.92E-03 | BP | phosphorylation |
| GO:0006364 | 9 | 1.83 | 3.32E-02 | BP | rRNA processing |
| GO:0006813 | 14 | 1.73 | 1.86E-02 | BP | potassium ion transport |
| GO:0007165 | 39 | 1.62 | 5.52E-04 | BP | signal transduction |
| GO:0016192 | 36 | 1.40 | 2.42E-02 | BP | vesicle-mediated transport |
| Non-conserved group |  |  |  |  |  |
| GO:0009611 | 4 | 5.21 | 0.00E+00 | BP | response to wounding |
| GO:0017000 | 4 | 5.21 | 0.00E+00 | BP | antibiotic biosynthetic process |
| GO:0043248 | 4 | 5.21 | 0.00E+00 | BP | proteasome assembly |
| GO:0006979 | 5 | 3.72 | 1.54E-03 | BP | response to oxidative stress |
| GO:0006013 | 3 | 3.13 | 2.76E-02 | BP | mannose metabolic process |
| GO:0006631 | 3 | 3.13 | 2.76E-02 | BP | fatty acid metabolic process |
| GO:0007021 | 3 | 3.13 | 2.76E-02 | BP | tubulin complex assembly |
| GO:0006855 | 8 | 2.98 | 1.45E-03 | BP | drug transmembrane transport |
| GO:0006635 | 4 | 2.60 | 3.55E-02 | BP | fatty acid beta-oxidation |
| GO:0006508 | 56 | 1.53 | 1.22E-03 | BP | proteolysis |

GO categories are shown only if they include at least three genes. Enrichment scores were calculated by dividing the proportion of target gene set classified into the indicated category by the proportion of those in the genome background. BP: Biological process.

Table S9. DNA and RNA sequencing library list and data accession numbers.

| Strain | Sample type | Sequencing platform | Accession number | Note | Collected location and date | Generation difference of two repeats |
| --- | --- | --- | --- | --- | --- | --- |
| Genomic DNA reads used in assembly |  |  |  |  |  |  |
| Dd1 | White | PacBio Sequel/Gel-plus 10K | SRR9721675 | MAC |  |  |
| Genomic DNA reads used for re-mapping |  |  |  |  |  |  |
| Dd1 | White | NextSeq/Paired-end 300 | SRR9720325 | MAC | Hitachiota-city, Ibaraki pref, Japan, 1995 |  |
| Dd1 | White | NextSeq/Paired-end 300 | SRR9720324 | Whole cell |  | 8 |
| KM2 | White | NextSeq/Paired-end 300 | SRR9721673 | MAC | Matsue-city, Shimane pref, Japan, 1985 |  |
| KM2 | White | NextSeq/Paired-end 150 | SRR9721674 | Whole cell |  | 148 |
| HK1 | White | NextSeq/Paired-end 150 | SRR9721671 | Whole cell | Chigasaki-city, Kanagawa pref, Japan, 1990 |  |
| HK1 | White | NextSeq/Paired-end 150 | SRR9721672 | Whole cell |  | 84 |
| STL3 | White | NextSeq/Paired-end 150 | SRR9721676 | Whole cell | Shimabara-city, Nagasaki pref, Japan, 1988 |  |
| STL3 | White | NextSeq/Paired-end 150 | SRR9721677 | Whole cell |  | 84 |
| DK1 (Progeny of Dd1 and KM2) | White | NextSeq/Paired-end 150 | SRR10803125 | Whole cell | 25-Nov-2016 |  |
| DK1 (Progeny of Dd1 and KM2) | White | NextSeq/Paired-end 150 | SRR10803124 | Whole cell |  | 100 |
| DK2 (Progeny of Dd1 and KM2) | Green | NextSeq/Paired-end 150 | SRR11177409 | Whole cell | 15-Aug-2019 |  |
| RNAseq library |  |  |  |  |  |  |
| Dd1 | Green | NextSeq/Paired-end 150 | SRR9720326/SRR9720327/SRR9720328 | Whole cell |  |  |

Table S10. Primer list used in qPCR and Southern blot and F1 progeny validation.

| Target gene | Primer name | Sequence | Primer name | Sequence |
| --- | --- | --- | --- | --- |
| 000169F_H1.43 | 169F.43 F1 | TCGATCCTCTTGATGGAAGTTCA | 169F.43 R1 | ACCATATAGACAACAAAGTGCAGAAATT |
| 000086F_H1.45/000086F_H1.46 | 86F.45 F1 | GCCTATCTTGCTGCTGGTTTG | 86F.45 R1 | ATTGCGCATTTGCTCTCACA |
| 000081F_H1.42 | 81F.42 F1 | CCGGATTGGATGCTTAGGTGTA | 81F.42 R1 | AGGGTGATCAATATGGACGAGAA |
| 000012F_H1.5 | 12F.5 F1 | GCTGTTCTATGGCAGTCTGGTTT | 12F.5 R1 | TTTTCCATTAGGTGGATGATCAACT |
| 000287F_H1.6 | 287F.6 F1 | GAGAGAGCACAGCCATTAGCAA | 287F.6 R1 | CATTAGCACCCTTCTTGATTTGTTT |
| 000040F_H1.50 | 40F.50 F1 | TTTCAAATGGGAAATGCCTAAGTT | 40F.50 R1 | GTATCTGGTTTCATATGCCATGCT |
| 000482F_10633-13372_H1.1 | 482Fs.1 F1 | CTCAAGATACAGGCTCATTAGTGAATG | 482Fs.1 R1 | CTTATGCCCATACAGATATTGTGAATC |
| 000041F_H1.14 | 41F.14 F1 | GAAACTTCAGCTAAAGATGGAACAAA | 41F.14 R1 | TGGGTCTTGAGTAACTTATGAATTGC |
| 000169F_H1.39 | 169F.33 F1 | TGGAAGCACGCTTGCAGATA | 169F.33 R1 | AACCTTTTCCAGTTACCGTGTGTAT |
| 000107F_H1.69 | 107F.69 F1 | TCCCCTTGCCATGTTTCTTG | 107F.69 R1 | GGCCTACGTATACTGGAGAATGGT |
| 000023F_H1.56 | 23F.56 F1 | GCATGATACCTAAGCGTACCACAA | 23F.56 R1 | ACAAGAGGCAACGCACAAAA |
| 000022F_H1.26 | 22F.26 F1 | GTGTATTTTAGCAACCATCGCATT | 22F.26 R1 | AGCAGCAGAAGGCCAATAACA |
| 000009F_H1.42 | 9F.42 F1 | ATTCACCCACGCCAACCAT | 9F.42 R1 | AGCAATCACTTATGCAGGGCTAT |
| 000001F_H1.83 | 1F.83 F2 | AATTTGTCCCTTTTGCATCAGAGTA | 1F.83 R2 | TCCAAGAACCAATCAGGCTTGT |
| 000025F_H1.23 | 25F.23 F1 | GTCGATTGCCCATGCTTCA | 25F.23 R1 | GAGATTGTTGATTTCTTGAGTTATTGGA |
| 000125F_H1.32 | 125F.32 F1 | CGAGTGTAGCAACACCAAGCA | 125F.32 R1 | TTTCTCCCATTAAGTCAAACCTTGA |
| 000413F_H1.4 | 413F.4 F1 | TATGGTGCTCCTCCTGTTTAAGAA | 413F.4 R1 | ATGCAGAAACCTAAGAATACAAGAACAA |
| 000010F_H1.39 | 10F.39 F1 | TAACCGATGTATGATGCGAAATG | 10F.39 R1 | TCCTGGGAACGGTTATTTTCC |
| For Southern blot |  |  |  |  |
| *P. bursaria* mitochondrial gene: COX1 | mtDNA F2 | GCATTTTTAAGATCCACTAATTG | mtDNA R1 | GGGGTAACAATGGCTAACATTC |
| *Paramecium bursaria* Chlorella virus-1 gene:A034R | PBCV-3 F1 | GTAAGATTCTCGTATTCGTCGC | PBCV-3 R1 | CAACCAATGGTATCACTTCTCC |
| 000334F_H1.12 promoter region | Promoter F1 | CCTTTAGAATAGCCAAAAAAATCAATATACAAGC | Promoter R1 | CTTTCAAAATTTTTAAGGTAATTTTATAAAAGAA |
| For progeny validation |  |  |  |  |
| 000029F_H2.20 | CAL-F1 | GGCTGAATAACTCACAGAAG | CAL-R2 | GTGTCCATCTCCATCAATATCAG |
| 000000F_H1.4 | 000000F_F1 | CCTTCTGGATGTCCTATTATTTAC | 000000F_R1 | GCAAGGTCAGTTCATATGTGTTTAGG |
| 000002F_H1.5 | 000002F_F1 | CAGAAGTAGATCCTATGATGGG | 000002F_R1 | GAACCAGTATTTGAAGGAGATG |
